# Moonlighting Peptides with Emerging Function

**DOI:** 10.1371/journal.pone.0040125

**Published:** 2012-07-13

**Authors:** Jonathan G. Rodríguez Plaza, Amanda Villalón Rojas, Sur Herrera, Georgina Garza-Ramos, Alfredo Torres Larios, Carlos Amero, Gabriela Zarraga Granados, Manuel Gutiérrez Aguilar, María Teresa Lara Ortiz, Carlos Polanco Gonzalez, Salvador Uribe Carvajal, Roberto Coria, Antonio Peña Díaz, Dale E. Bredesen, Susana Castro-Obregon, Gabriel del Rio

**Affiliations:** 1 Biochemistry and Structural Biology Department, Instituto de Fisiología Celular, Universidad Nacional Autónoma de México, México D.F., México; 2 Biochemistry Department, Facultad de Medicina, Universidad Nacional Autónoma de México, México D.F., México; 3 Centro de Investigaciones Químicas, Universidad Autónoma del Estado de Morelos, Cuernavaca Morelos, México; 4 Department of Developmental Genetics and Molecular Physiology, Instituto de Biotecnología, Universidad Nacional Autónoma de México, Cuernavaca Morelos, México; 5 Molecular Genetics Department, Instituto de Fisiología Celular, Universidad Nacional Autónoma de México, México D.F., México; 6 Buck Institute for Age Research, Novato, California, United States of America; 7 Department of Neurology, University of California San Francisco, San Francisco, California, United States of America; Semmelweis University, Hungary

## Abstract

Hunter-killer peptides combine two activities in a single polypeptide that work in an independent fashion like many other multi-functional, multi-domain proteins. We hypothesize that emergent functions may result from the combination of two or more activities in a single protein domain and that could be a mechanism selected in nature to form moonlighting proteins. We designed moonlighting peptides using the two mechanisms proposed to be involved in the evolution of such molecules (*i.e*., to mutate non-functional residues and the use of natively unfolded peptides). We observed that our moonlighting peptides exhibited two activities that together rendered a new function that induces cell death in yeast. Thus, we propose that moonlighting in proteins promotes emergent properties providing a further level of complexity in living organisms so far unappreciated.

## Introduction

Hunter-killer peptides are molecules designed to induce cell death on selected eukaryotic cells [1]. The specificity of their action is based on a hunter moiety, which is a peptide that acts as ligand for a receptor expressed on targeted cells. The induction of cell death is achieved by a killer moiety, which is a mitochondrial active peptide capable of inducing the release of cytochrome-C, caspase activation and ultimately apoptosis. These two moieties are coupled by a linker-peptide, commonly a glycine-glycine peptide. A feature of killer peptides is that they are not toxic to eukaryotic cells, unless these are internalized. Thus, it is assumed that the induction of cell death by hunter-killer peptides is achieved when the killer-peptide is internalized by means of receptor-mediated endocytosis of the hunter-peptide [2]. While these peptides have shown to be effective in treating cancer [3], [4], [5] or obesity in animal models [6], there is still much room for improvement [7] provided that more studies are conducted about their mechanism of action.

In the current work we aim to create new hunter-killer peptides against *Saccharomyces cerevisiae*, as a further step in understanding their mechanism of action. In this report we focus on the design of hunter-killer peptides that have the two activities in a single functional domain.

Many proteins are known to have more than one activity in a single domain. The property of proteins having multiple activities is sometimes referred to as moonlighting [8], [9]. Moonlighting proteins and peptides presenting more than one activity in a single domain differ from proteins that have multiple activities in multiple domains [10], implying different evolutionary constraints (see below). Additionally, many non-globular moonlighting proteins and peptides exhibit a disordered state [11]. These proteins with a disordered structural state are commonly referred to as natively unfolded proteins (NUP), intrinsically unstructured or intrinsically disordered proteins [12]. This structural plasticity has advantageous consequences, especially in eukaryotic cells, where an increased phenotype is observed without concomitantly increasing the genotype [13].

Based on these features of moonlighting proteins, different evolutionary mechanisms have been proposed to explain the presence of multiple activities in single domain proteins. One mechanism implies that during the evolution of globular moonlighting proteins, the large unused surfaces have been used for accommodating additional activities; in the second mechanism, the lack of a constrained structure in NUP allows these proteins to perform different activities in different environments through a conformational selection mechanism [14]. In both cases, the addition of a new activity to an existing active-protein has been proposed to lead to an “adaptive conflict”; such conflict refers to the observation that most protein mutants might improve one activity while reducing the other [15]. The engineering of moonlighting peptides could provide further insight into the evolution of moonlight proteins yet, such engineering need to deal with the adaptive conflict. We propose that the combination of multiple activities into one protein domain may generate new function(s). After all, biological systems display emergent properties [16] and moonlighting proteins might be an important, but so far unappreciated mechanism of emergent functionalities in cells.

To test the idea that combining two or more activities in a single functional domain may render emergent properties in peptides, we used a 13-residue long peptide, the α-pheromone from *S. cerevisiae*, as a hunter peptide. From this natively unfolded peptide [17], we designed several moonlighting peptides by adding residues to the α-pheromone to reproduce the physicochemical properties of killer peptides; this rendered 19-residue long peptides. The killer activity requires an alpha-helical conformation, which is a conformation not observed in the α-pheromone peptide [18], [19], [20]. Finally, these two activities (pheromone and selective antibacterial activities) combined in a single functional domain (selective antibacterial peptide) rendered peptides with a new function: the killing of *S. cerevisiae* cells. We named these Iztli peptides, by reference to the Aztec’s mythology god of sacrifices; by extension, we refer to the hypothesis that leads to the design of these peptides (*i.e*., merging two activities into a single functional domain may render emergent properties) as the Iztli hypothesis.

## Results and Discussion

To facilitate testing for the new activity added to the α-pheromone, we considered that the killing activity should be triggered in an environment different to the α-pheromone receptor; in our case, that would be the bacterial or mitochondrial membrane.

Combining a ligand peptide (hunter) with a selective antibacterial peptide (killer) is known to induce cell death in mammalian cells by hindering mitochondria function [7]. However, we will show that damaging mitochondria function or signaling by the pheromone peptide alone is not enough to kill yeast cells. On the other hand, it has been reported that mutating the α-pheromone in either the N- or C- terminus renders a non-functional peptide [21]. Thus, our approach to add a new activity to the pheromone by adding extra residues in either the N- or C- terminus of the pheromone constitutes an example of an adaptive conflict paradox.

**Figure 1:**
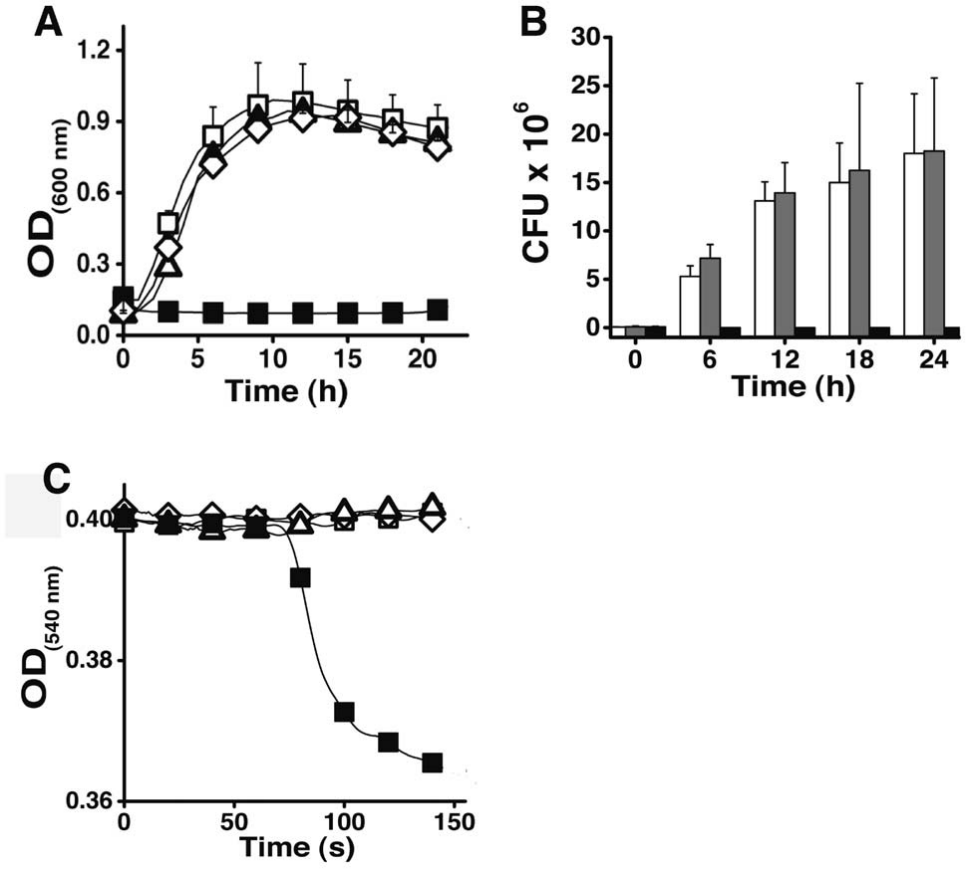
Antibacterial and mitochondrial swelling activities of Iztli peptides. The activity for Iztli peptide IP1 (20 µM) (▪ in **A**, black bars in **B**) against the bacteria *E. coli* (DH10B) was tested in two ways: following the optical cell density of the culture at 600 nm (**A**) and counting the colony forming units (CFU) (**B**). Three controls are included: LB with no peptide (**▪** in **A**, white bars in **B**)**,** α-pheromone (25 µM; ▪ in **A**, gray bars in **B**) and the six residues at the N-terminus of IP1 (fIP1) (119 µM; ⋄ in **A**)**.** The results of 4 experiments are presented for plots in **A** and **B**. The bars represent standard deviations. Mitochondrial swelling was followed at 540 nm (**C**) in the presence of Iztli peptide IP1 (▪ 117 µM); three controls were used: distilled water (**▪)**, α-pheromone (▪ 160 µM) and fIP1 (⋄ 100 µM), Polyethylene glycol 3.4 kDa was added at 180 seconds. Only the IP1 data is shown here; the 3 other IP peptides display similar activity than IP1.

**Figure 2:**
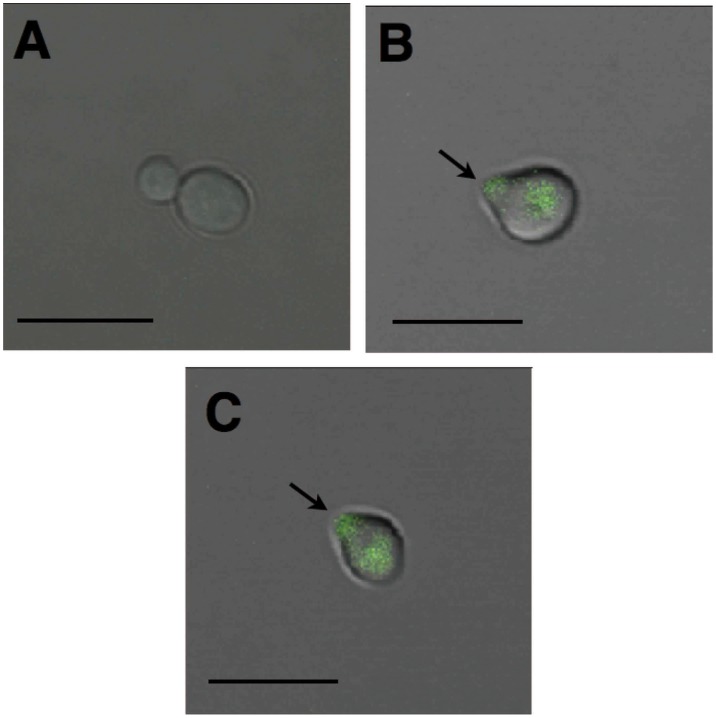
Pheromone activity of Iztli peptides. The pheromone-like activity of Iztli peptide IP1 was determined in two ways: detecting the activation of Fus1-GFP and observing the Shmoo phenotype. The image presents MatA cells in media without pheromone (A) and those presenting both Shmoo phenotype (small protrution on the cells indicated with the arrow) and the Fus1-GFP fluorescence observed after 1 hr of induction in the presence of either the α-pheromone (B) or the Iztli peptide IP1. (C) This is a compose image from DIC and fluorescence microscopy. Images were generated using a confocal microscope Olympus FluoView FV1000 with a magnification of 60×. The scale line is 5 µm.

To incorporate the selective antibacterial activity to the α-pheromone, we modified its physicochemical properties (AGADIR score, isoelectric point and hydrophobic moment) to match those of known selective antibacterial peptides by adding every amino acid residue on either N- and/or C- terminus of the α-pheromone (for the strategy followed to design these peptides, please refer to section II in the Supporting Information S1; see Table S2 for the list of peptides tested and Figures S10, S11, S12, S13, S14 and S15 for the evaluation of our designs). Only peptides adding 6 extra residues on the N-terminus of the α-pheromone rendered peptides with predicted physicochemical properties characteristic of known selective antibacterial peptides; these residues locate in the linker region connecting the multiple copies of the α-pheromone in the protein precursor [22]. Figure 1 shows that indeed Iztli peptides are able to kill bacteria (Figure 1A and 1B) and to evoke mitochondrial swelling (Figure 1C). We also observed that our Iztli peptides maintain pheromone-like activity (Figure 2) similar to that of the original α-pheromone. Thus, Iztli peptides exhibit two activities (pheromone-like activity and antibacterial activity) in a single functional domain (antibacterial domain).

**Figure 3:**
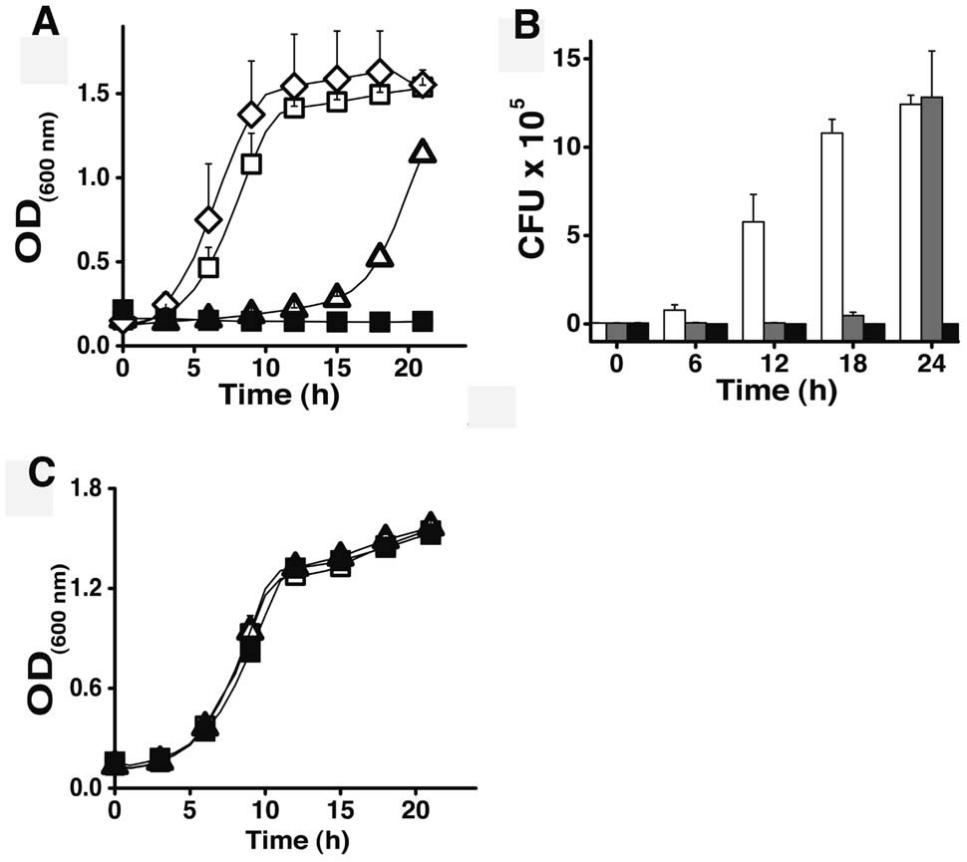
Antifungal activity of Iztli peptides. The activity of the Iztli peptide IP1 (10 µM) (▪ in **A**, black bars in **B**) against the MatA cells from *Saccharomyces cerevisiae* (BY4741) is presented in two formats: Optical cell density (**A**) and CFU (**B**). Cells were grown in YPD. We used three controls: No peptide (**▪** in **A**, white bars in **B**), α-pheromone (10 µM) (▪ in **A**, gray bars in **B**) and the six residues at the N-terminus (fIP1) (60 µM) (⋄ in **A**). The results of 4 experiments are presented for plots in **A** and **B**. The error bars represent standard deviations. The strain BY4741 Δ*STE2* lacking the receptor for the α-pheromone was tested against the Iztli peptides (**C**): IP1 ▪ 10 µM, IP2 ▪ 10 µM for antifungal activity. For these experiments we used no peptide **▪** and α-pheromone ▪ (10 µM) as controls.

Then we tested the ability of Iztli peptides to kill *S. cerevisiae*, and observed that indeed these peptides have antifungal activity (see Figure 3A and 3B). Note that such variants of the α-pheromone are unlikely to be found in *S. cerevisiae* cells; however, we envision that other yeast species (*e.g*., *Candida glabrata*, which produces a pheromone similar to the α-pheromone [23]) could select for this class of peptides to outcompete *S. cerevisiae*.

As expected from the embedded pheromone activity of Iztli peptides, the killing activity depends on the presence of the α-pheromone receptor, Ste2p. Indeed, a mutant strain lacking Ste2p was resistant to Iztli peptides (see Figure S1). Furthermore, Iztli peptides conserved the pheromone-binding mode to Ste2p, as can be observed by introducing mutations that have been previously reported to increase (Gln5Ala, Pro8Ala) or reduce (His2Ala, Gly9Ala, Tyr13Ala; numbering referring to the α-pheromone sequence) the affinity of the α-pheromone for the Ste2p receptor [24] (see Figure S2). Finally, the Iztli hypothesis implies that the activity to swell mitochondria and to kill *S. cerevisiae* cells expressing the Ste2p receptor should not reside solely on the 6 residues added to α-pheromone; instead the pheromone-like activity, the antibacterial activity and the capacity to kill yeast cells should be present in a single functional domain. To test this, we treated isolated mitochondria and yeast cells with the six residues at the N-terminus of IP1 (fIP1); this peptide did not exhibit any activity (Figures 1 and 3).

These physiological data (Figures 1, 2, 3 and Figures S1 and S2) indicate that Iztli peptides possess pheromone-like activity, antibacterial activity (or to swell mitochondria) and the capacity to kill yeast cells, all embedded in a single functional domain.

### Structural Properties of Iztli Peptides

**Figure 4:**
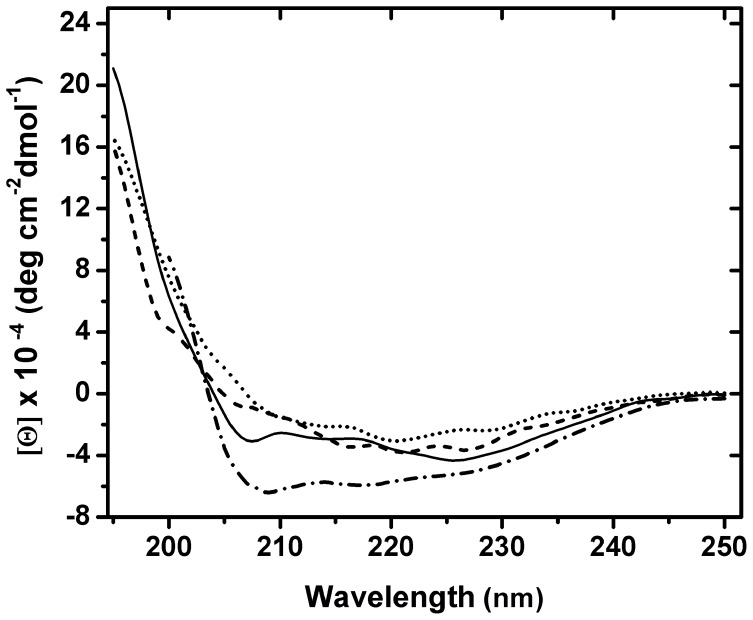
Secondary structure of Iztli peptides in TFE. The observed conformation of the four Iztli Peptides (IP1 dashed-dotted line, IP2 solid line, IP3 dashed line and IP4 dotted line) in 50% Trifluroethanol is shown. CD spectra of each peptide in water were subtracted from the spectra of corresponding peptide in TFE.

According to our design, Iztli peptides should be able to form an α-helical structure (see Supporting Information S1). The α-pheromone is known to be unstructured both in water and in trifluoroethanol (TFE) [19], a structure-inducing solvent. Additionally, nuclear magnetic resonance (NMR) studies had shown that in the presence of membranes, the α-pheromone has five residues in a helical turn at the N-terminus [25]; our peptides were designed to adopt an alpha-helical structure at the N-terminus, thus potentially extending the helical structure adopted by the α-pheromone (Figure S3). To test this, we analyzed the circular dichroism (CD) spectra of our peptides in water and in the presence of TFE. Far-UV CD spectra of all four peptides shows marked differences between water and 50% TFE (Figure S4). In water, the CD spectra show no evidence of α-helix, suggesting that the peptides are essentially disordered. Specifically, each peptide had a larger CD intensity within the 215–230 nm wavelength spectra region. A weaker positive band at ∼217–230 nm and a strong negative band around 200 nm are consistent with a polyproline type II (PP_II_) structure; random coil structures are characterized by a strong negative band below 200 nm and a positive one around 218 nm. However, the side chains of aromatic amino acid residues also contribute to the electronic absorption in the far-UV region and positive bands at 197 nm, 217–220 nm and 235 nm have been attributed to Phe, Tyr and Trp side chains [19]. Since, all peptides have two Trp, one Tyr and one or two Phe residues, the TFE-induced helical signal in the far-UV CD spectra of the peptides would be superimposed to the band around 220–230 nm of the absorption contributions of their aromatic side chains (Figure S4). Thus, we subtracted the absorbance of the peptides in water from the ellipticity values of the CD spectra of peptides in TFE and this differential spectra clearly shows, that Iztli peptides adopts primarily α-helical conformation (see Figure 4). The original and subtracted CD spectra of peptides in TFE were deconvoluted using three different analysis computer programs (see Methods) to obtain the percentages of α-helix. The IP1 and IP2 peptides in TFE were estimated to contain 75–90% α-helix, while the IP3 and IP4 peptides up to 70%.

To confirm the CD observations we determined the crystal structure of IP2, which was the only Iztli peptide that provided crystals useful for diffraction experiments. The IP2 structure was solved at 3.4Å resolution using a seleno-derivative (Met11-SelenoMet11) that retained biological activity (see Figure S5). An alpha-helical structure with up to 3 turns was inferred (Figure S6 in alpha-carbons) that most likely lays in the N-terminus of the peptide: *i.e*., sequence propensity to form such structure is predicted on the N-terminus and the selenium atom in the Met11-SelenoMet11 variant located at the C-terminus was highly flexible in the crystal (see Table S3 and Figure S7). However, it was not possible to refine the molecular model of the peptide due to a crystal twinning issue (see Supporting Information S1, section I.10), thus we further studied with NMR spectroscopy the structural properties of this peptide.

A two-dimensional total correlated spectroscopy (2D-TOCSY) spectrum was used for the identification of spin systems whereas a two-dimensional nuclear Overhauser effect (2D-NOESY) spectrum was used to check space connectivities. We found more NOEs cross-peaks (see Figure S8) in the TFE spectra and sequential HN connection for the N-termini residues (data not shown), which were not observed in the H_2_O spectrum. These observations are consistent with the CD and X-ray data, confirming our conclusions on the existence of an α-helix at the N-termini.

These structural data (Figure 4 and Figures S3, S4, S5, S6, S7 and S8) indicate that Iztli peptides have the capacity to switch from a random coil to an alpha-helical structure when they are transferred to a non-aqueous solvent. This is consistent with the behavior of disordered moonlighting proteins, which present multiple conformational states (disordered and globular) when they are exposed to different environments.

### Emergent Function of Iztli Peptides

Finally, we analyzed the emergent function of Iztli peptides. Like hunter-killer peptides, Iztli peptides combine an antibacterial peptide (killer peptide) with a ligand peptide (hunter peptide). The killer peptide induces mitochondrial damage; such damage in mammalian cells leads to apoptosis. However, it could be expected that mitochondria damage in yeast cells does not necessarily lead to apoptotic cell death; likewise, pheromone signaling at physiological concentrations does not kill all yeast cells (see below). Thus, the observed killing of yeast by Iztli peptides should require more than the independent activities of the hunter (pheromone) or the killer peptides.

In agreement with this idea, it should be noted that:

1. Cell death induced by Iztli peptides requires the Ste2p transduction system, not simply the Ste2p receptor, since a null mutant of the STE4 gene (which encodes the β subunit of the heterotrimeric G protein coupled to Ste2p in the *S. cerevisiae* mating pathway [26]) was resistant to the killing activity of Iztli peptide 1, IP1 (see Table S1 and Figure S1).
2. The absence of the apoptosis-inducing factor (AIF1), the meta-caspase (MCA1) and 25 other apoptotic related genes do not affect the killing by Iztli peptides (Table S1 and Figure S1).
3. More than 5 times pheromone than Iztli peptides is required to kill *S. cerevisiae* mating type a cells (MatA): α-pheromone induces partial cell death at >50 µM [27] while our Iztli peptides induced complete cell death at 10 µM (see Figure 3), suggesting the pheromone activity in the Iztli peptides alone cannot explain the observed cell death.
4. The α-pheromone at high concentrations may induce a non-apoptotic cell death mechanism that exhibited some dependency on mitochondrial respiration in MatA cells [28]. However, in such cases, cells maintained mitochondrial respiration at all time and only 25% of the cell population was killed by the α-pheromone. Iztli peptides eliminate mitochondrial respiration in vivo (Figure S9) and kill all cells, as no growing cells remain after 24 hours (see Figure 3A and 3B).

These data confirm that cell death induced by Iztli peptides affects mitochondria respiration and depends on the signaling pathway of Ste2p, albeit it is independent of apoptosis. Thus, it is possible that while yeast cells may survive to either cell cycle arrest induced by the α-pheromone or to respiratory-deficient mitochondria, the combination of these two conditions pose critical conditions that the cell cannot surpass. To quantify the level of interaction between these two activities in cell yeast survival, we estimated the relative amount of surviving cells (colony forming units, CFUs) in non-breathing ρ0 yeast cells (100%), in cells exposed to the α-pheromone (100%) and our Iztli peptide (0.00025%) based on the observed surviving rate in wild-type cells (see Supporting Information S1, section I.6). If swelling mitochondria and arresting the cell cycle would have an independent effect, the survival rate would be at most the sum of the individual effects (100%); yet, the observed survival rate in the presence of Iztli peptides is smaller. Thus, Iztli peptides present an emergent function because it is not simply the result of adding the effect of the two activities alone.

The existence of moonlighting proteins in energy-restricted conditions has been proposed to present an advantage over the maintenance of multiple copies of genes. However, in polyploid cells or cells cultivated in the lab in rich media during many years (*e.g., S. cerevisiae*), such energetic restriction does not prevail and yet moonlighting proteins are present [29]. Alternatively, moonlighting proteins have been proposed to be important in coordinating related cellular activities; consistent with this idea is the existence of moonlighting proteins that combine related activities (*e.g*., LON is a mitochondrial ATP-dependent protease that is also a mitochondrial chaperone [30]), although this relationship is not clear for all moonlighting proteins (*e.g.*, the band 3 protein of the red blood cell plasma membrane is an anion exchanger and also regulates the rate of glycolysis [31]). To solve these discrepancies, we propose that moonlighting in single-domain proteins occurs only when the combined activities render an emergent function. That is, the use of single-domain moonlighting proteins represents a significant challenge for screening the appropriate sequence that can harbor multiple activities (adaptive conflict) in comparison with moonlighting proteins presenting different activities in different domains. Thus, we propose that single-domain moonlighting proteins are selected over multi-domain when they present new activities beyond those obtained by combining different activities in different domains.

Our Iztli peptides have not been designed to treat cells that represent a health problem, as in the case of hunter-killer peptides. However, we envisioned potential uses for our peptides in basic research, for example: a) eliminating MatA cells in a population (*e.g*., selection of *S. cerevisiae* mating type α cells (MatAlpha) haploids), b) study the mechanism of action of hunter-killer peptides, c) structure-function studies of moonlighting peptides. In addition, it is clear that the selective antibacterial activity of Iztli peptides has potential clinical applications.

In summary, we have shown that altering the N-terminus region of the mating α-pheromone from *S. cerevisiae* to display selective antibacterial activity (and for swelling mitochondria), rendered peptides capable to induce yeast cell death. Swelling of mitochondria and the pheromone activities in these peptides encompass two conformations that can be switched by the environment. Finally, our designs show that the ability of proteins to combine multiple activities in a single domain may lead to the acquisition of novel functions. This emergent function might be selected in nature, thus eliminating the “adaptive conflict” in the evolution of moonlighting proteins.

## Materials and Methods

### I.1 Strains

The *S. cerevisiae* strains used for the identification of critical genes involved in the killing activity of the Iztli peptides are listed in Table S1.

To evaluate the antibacterial activity of the Iztli peptides, the *Escherichia coli* DH1OB strain, Δ(mrr-hsd RMS-mcrBC) mcrA recA1 was used.

### I.2 Peptides

The mating α-pheromone from *S. cerevisiae* was obtained from Sigma-Aldrich (catalog number T6901). Anaspec, Inc. (USA) synthesized the Iztli peptides (see Table 1). The company verified the purity of these peptides using High-performance liquid chromatography (HPLC) and mass spectrometry (see Table S4 and Figures S16 and S17); optimized Fmoc and Boc methodologies were employed for peptide syntheses with free N- and C- termini. Each peptide (2 mg) was diluted in 1 mL of water and keep at −80°C as stock solutions. The actual concentrations of these solutions were determined at 280 nm using a Nanodrop equipment (Thermo Scientific, USA). All peptides used in this study included only L-amino acids.

**Table 1:** Sequences of Iztli peptides.

Name	Sequence
α-pheromone	WHWLQLKPGQPMY
IP1	KFLNRFWHWLQLKPGQPMY
IP2	RRLKDFWHWLQLKPGQPMY
IP3	KFWKRFWHWLQLKPGQPMY
IP4	RKLQKFWHWLQLKPGQPMY
fIP1	KFLNRF
IP1-His8Ala	KFLNRFWAWLQLKPGQPMY
IP1-Gln11Ala	KFLNRFWHWLALKPGQPMY
IP1-Pro14Ala	KFLNRFWHWLQLKAGQPMY
IP1-Gly15Ala	KFLNRFWHWLQLKPAQPMY
IP1-Tyr19Ala	KFLNRFWHWLQLKPGQPMA
IP2SEM	RRLKDFWHWLQLKPGQP(SeMet)Y

### I.3 Minimum Inhibitory Concentration

Lyophilized peptides were solubilized in sterile milli-Q water to a final concentration of 4 mg/ml except α-pheromone-PH(Cecropin)1, α-pheromone-PH(CeMa)1, PH(SCAP*)2 2 mg/ml and PH(SCAP*)5 20 mg/ml. Serial dilutions of peptides were prepared in sterilized water. These concentrations are reported as the dry weight of each peptide. To determine the actual concentration of these peptides in solution, we estimated the molar absorption coefficient from the absorbance of each peptide at 214 nm [32] using a spectrophotometer HP8452, kindly provided by Dr. Armando Gomez-Puyou. Then, the concentration of each peptide was calculated as: C = A/(L*MAC); where C: concentration (M), A: absorbance (arbitrary unit), L: length of the path of the spectrophotometer cell (cm) and MAC: the molar absorption coefficient (M^−1^cm^−1^).

The antibacterial activity of the synthetic peptides was examined in sterile 100-well plates (HC2 Pat. Pend., Finland) in a final volume of 200 µl as follows: aliquots (25 µl) of a suspension containing bacteria at a concentration of 10^5^ CFU/mL in LB medium were added to 75 µl of water containing the peptide. The mixture was completed by the addition of 100 µl of 2×-concentrated LB medium. The plates were incubated at 37°C with constant orbital shaking for 10 h (at a rate specified by the equipment provider as “High”). Microbial growth was automatically determined by reading every 60 minutes the optical density at 600 nm with an OY Microplate Reader (Bioscreen C, Finland).

The minimum inhibitory concentration (MIC) was determined as the lowest concentration where no visible growth occurred. At least three different concentrations were tested for each peptide to find the corresponding MIC. All MICs were determined from four independent experiments.

### I.5 Detection of Pheromone-like Activity on Iztli Peptides

MatA yeast cells (BY4741) grown for 24 hr in YPD media at 30°C were diluted to a final optical density of 0.4 at 600 nm. These cells were treated with 10 µM of the Iztli Peptide 1 for one hour. After that time, a sample of 10 µL was observed under the confocal microscope (Olympus FluoView FV1000) at 60× amplification. The images were reconstructed using the software FV viewer provided by the manufacturer of the microscope.

### I.10 Mitochondrial Swelling and Contraction

We isolated mitochondria as described in Supporting Information S1 (see section I.2). Swelling of mitochondria was determined as reported before [33]. Briefly, fresh mitochondria were diluted in 1 mL of 0.3 M mannitol, 5 mM MES, pH 6.8 (TEA) and 5 µL of ethanol. After 20 seconds, when optical density was stable, the peptides were added. The absorbance at 540 nm was recorded in a DW2 Aminco spectrophotometer (Olis, Inc, USA) in a split mode with magnetic stirring. A decrease in the absorbance was indicative of mitochondrial swelling. To determine the integrity of mitochondrial membranes, 10% of PEG 10 kDa (final concentration) was used to induce water release from mitochondria. Thus, a recovery of the initial values of O.D.540 measurement in the presence of PEG after peptide exposure indicated that mitochondria were swollen by the peptide and not disrupted.

### I.11 Measuring Antifungal Activity of Iztli Peptides

An early stationary culture for every essay was obtained from a single colony growing overnight in YPD medium. This culture was used to inoculate a fresh YPD medium to reach 0.04 O.D. in a total volume of 200 µL. Every peptide and strain were tested in 96 well plates, each well containing 200 µL, incubating at 30°C with shaking using a Bioscreen C (Oy Growth Curves Ab Ltd, Finland). The activity of these peptides on each strain was determined by following the cultures during 24 hrs and recording the O.D. every hour. Lack of change in O.D. measured during 24 hours was indicative of antifungal activity.

### I.14 Circular Dichroism

To determinate the structural conformation of the peptides, 0.3 mg of each Iztli peptide was dissolved in 1 mL of water or 50% trifluoroethanol. Circular Dichroism spectra were recorded on a JASCO J-715 Spectropolarimeter (Jasco Inc. U.S.A.) at room temperature using a 1 mm path length cell. Spectra were acquired from 195 to 250 nm. Ellipticity is reported as mean residue molar ellipticity and recorded in terms of molar elipticity [θ] (deg cm^2^ dmol^−1^). Estimation of the secondary structure content of the peptides was performed using CDPro [34], CDNN and KJ2D analysis software programs.

## Supporting Information

Figure S1 **Screening for resistant genes to Iztli peptides.** The area under the curve (AUC) for each strain of *S. cerevisiae (BY4741)* harboring a gene deletion (referred by the ORF name) tested against IP1 (10 µM) was calculated and charted. The AUC control (0.09) is the value of the IP1 (10 µM) against *S. cerevisiae WT (BY4741).*
(TIF)

Figure S2 **Antifungal activity of Iztli peptides mutated in the recognition sequence.** The activity of Iztli peptide 1 mutated in: His8Ala ▪ (2 µM) Gly11Ala ▪ (2 µM), Pro14Ala ▾ (2 µM), Gly15Ala**⧫**(2 µM) and Tyr19Ala ▪(2 µM) were tested against *S. cerevisiae (BY4741)*, as a control the original IP1 ▪ (2 µM) was used.
(TIF)

Figure S3 **Secondary structure prediction of Iztli Peptides.** The SOPMA method was used to generate the image presented [35]. Predictions were generated using the default parameters for SOPMA. Iztli peptides have a larger propensity to form an alpha-helix at the N-terminus and random coil at the C-terminus.
(TIF)

Figure S4 **Circular dichroism of Iztli peptides**. Far-UV CD spectra of IP1 (A), IP2 (B), IP3 (C) and IP4 (D) in water (open symbols) or 50% TFE (closed symbols) are shown. The CD spectra were recorded at 25°C using 0.3 mg/mL (158 µM) of each peptide.
(TIF)

Figure S5 **Antifungal and antibacterial activity of IP2SEM.** The activity of the seleno methionine derivate of IP2 was tested against A) *S. cerevisiae (BY4741),* IP2SEM ▪ (10 µM), using like controls YPD without peptide **▪** and IP2 ▪(10 µM) and B) *E.coli (DH10B),* IP2SEM ▪ (17 µM), like controls were used LB without peptide **▪** and IP2 ▪(17 µM).
(TIF)

Figure S6 **Asymmetric unit of the crystal structure of Iztli peptide 2.** The crystal structure of Iztli Peptide 2 was solved at 3.4 Å resolution. Only the alpha-Carbon atoms are presented. The asymmetric unit contains 4 monomers between 11 and 15 residues, each presented as a ribbon of different color.
(TIF)

Figure S7 **View of the experimental electron density map.** Crystal structure of the SeMet derivative Iztli peptide 2. The map is represented as a blue mesh, contoured at 1.4 r.m.s.d.
(TIF)

Figure S8 **NMR data of IP2.** Expanded region of an overlay of 2D-TOCSY (black) and 2D-NOESY (red) spectra of IP2 in H2O a) and 80% TFE b), illustrating the effect of TFE in the inter-residues NOEs cross-peaks. An increase in the number of inter-residues NOEs cross-peaks in 80% TFE is observed and indicated by arrows and one-letter code for the corresponding amino acid residues. The spectra were recorded on a 700 MHz Varian at 298 K.
(TIF)

Figure S9 **In vivo mitochondrial effect by Iztli peptides.** Oxygen consumption of isolated mitochondria of *S. cerevisiae (BY4741)* was measured in presence of Iztli peptide IP1 ▪ (58 µM) added 80 second after the start of the tracing; as an indicator of respiratory control, CCCP (1 mM final concentration) was added after 180 seconds. For these experiments we used YPD medium without peptide **▪**, α-pheromone ▵(10 µM) and fIP1 ⋄ (100 µM) as controls. Iztli peptides IP2, IP3 and IP4 showed a similar effect on mitochondria than IP1. The experiments were repeated three times and the image shows one representative result.
(TIF)

Figure S10 **De novo design strategy for selective antibacterial peptides**. The strategy described in this work for designing new selective antibacterial peptides is summarized in the figure as follows: a) The data needed for designing SCAP is indicated on top of the scheme. The arrows indicate the relationships between these data: the calculated physicochemical properties of known SCAP are compared with those of peptide sequences generated *in silico*. b) The specific data used in this study are in the middle of the scheme, that is, the calculated physicochemical properties (AGADIR score, isoelectric point and hydrophobic moment) of Cecropin and Magainin were compared with those obtained from libraries derived from KL and the α-pheromone. c) The new SCAP obtained are indicated at the bottom of the figure (Physicochemically homologous peptides).
(TIF)

Figure S11 **Selected designed SCAP from library 1 are not toxic on HFF cells**. The viability of human foreskin fibroblasts (HFF) cells in the presence of water (control), the IP1 and IP2 peptides for 72 hrs are shown. The image shows the living cells stained in green and dead cells stained in red. The images were generated using a magnification of 20×.
(TIF)

Figure S12 **Dendogram of designed SCAP obtained from library 2**. The 30 peptide sequences derived from the α-pheromone from *S. cerevisiae* that were identified as potential SCAP are shown in a dendogram. Each peptide sequence is presented as a node leaf in this representation.
(TIF)

Figure S13 **Designed SCAP from library 2 are not toxic on HFF cells**. The viability of human foreskin fibroblasts (HFF) cells in the presence of the IP1 or IP2 peptides is shown. The image shows the living cells stained in green and dead cells stained in red. The images were generated using a magnification of 10×.
(TIF)

Figure S14 **Amphipathic and cationic peptides (control peptides) are not toxic on HFF cells**. The viability of human foreskin fibroblasts (HFF) cells in the presence of water and the PH(SCAP*)1, PH(SCAP*)2, PH(SCAP*)3, PH(SCAP*)4, PH(SCAP*)5 and PH(SCAP*)6 peptides are shown. The images were taken 72 hrs after peptide addition (see Methods). The image shows the living cells stained in green and dead cells stained in red. The images were generated with a magnification of 10× for PH(SCAP*)1 and PH(SCAP*)2; 20× for the rest of the peptides.
(TIF)

Figure S15 **The physicochemical space of peptides**. The frequency of predicted values observed in every peptide of length 8 (20^8^ peptides) for A) Isoelectric point and B) hydrophobic moment are displayed. Note that in A) several pH values are not populated (*i.e*., these have a discrete distribution), while in B) every possible value of hydrophobic moment is found (*i.e*. these exhibit a continuous distribution).
(TIF)

Figure S16 **Quality control of Iztli peptides by HPLC.** ANASPEC Inc tested each one of the four Iztli peptides reported in this study: A) IP1≥95% purity. B) IP2>95% purity. C) IP3>95% purity. D) IP4>95% purity.
(TIF)

Figure S17 **Quality control of Iztli peptides by mass spectrometry.** The molecular weight of the four Iztli peptides was determined by ANASPEC Inc. using mass spectrometry: A) IP1∶2490.6 g/mol. B) IP2∶2501.9 g/mol. C) IP3∶2576.6 g/mol. D) IP4∶2485.9 g/mol.
(TIF)

Table S1 **Saccharomyces cerevisiae strains.**
(DOC)

Table S2 **Designed selective antibacterial peptides.** SCAPs designed in this study are presented indicating their names, sequence, predicted isoelectric point (IP) and helical hydrophobic moment (HM). The observed minimum inhibitory concentration for bacteria (MIC) and human cells (Toxicity) are indicated. For comparison, the SCAP used to match their physicochemical properties (Cecropin, Magainin, CeMa) and the α-pheromone are shown at the bottom of the table. Note that several of these peptides did not show any toxicity against bacteria or mammalian cells (*i.e*., PH(SCAP*)1.6 and the α-pheromone), therefore the corresponding concentration for the MIC or toxicity are preceded with “bigger than” symbol (>), indicating that possible toxic concentrations should be larger than those tested in this study. ND: not determined. (*) Data obtained from [36]. See section II.1 in this Supporting Information S1 for the rationale on the nomenclature of these peptides.
(DOC)

Table S3 X-ray data collection and refinement statistics. Values in parentheses are for the last resolution shell.
(DOC)

Table S4 Quality control data for Iztli peptide synthesis. Percentage Peak Area reported by ANASPEC Inc. using High-performance liquid chromatography (HPLC) for the four Iztli peptides.
(DOC)

Supporting Information S1 **Materials and methods of supplementary figures and tables.** The methods used to obtain the reported supplementary figures and tables are included in this document. Also, the corresponding references for these methods are included in this document.
(DOC)
