# Correction: Moonlighting Peptides with Emerging Function

**DOI:** 10.1371/annotation/fc687f18-ce8e-4704-87a5-d6807953204e

**Published:** 2013-10-23

**Authors:** Jonathan G. Rodríguez Plaza, Amanda Villalón Rojas, Sur Herrera, Georgina Garza-Ramos, Alfredo Torres Larios, Carlos Amero, Gabriela Zarraga Granados, Manuel Gutiérrez Aguilar, María Teresa Lara Ortiz, Carlos Polanco Gonzalez, Salvador Uribe Carvajal, Roberto Coria, Antonio Peña Díaz, Dale E. Bredesen, Susana Castro-Obregon, Gabriel del Rio

There were a number of errors in the legends for Figures 1, 3, S2, S5, and S9. The complete, correct Figure legends can be found here: http://www.plosone.org/corrections/pone.0040125.001.cn.tif

