# Supplementary figures and images for: Moonlighting Peptides with Emerging Function
Source: PLoS One. 2012 Jul 13;7(7):e40125. doi: 10.1371/journal.pone.0040125 (PMC3396687; doi:10.1371/journal.pone.0040125)

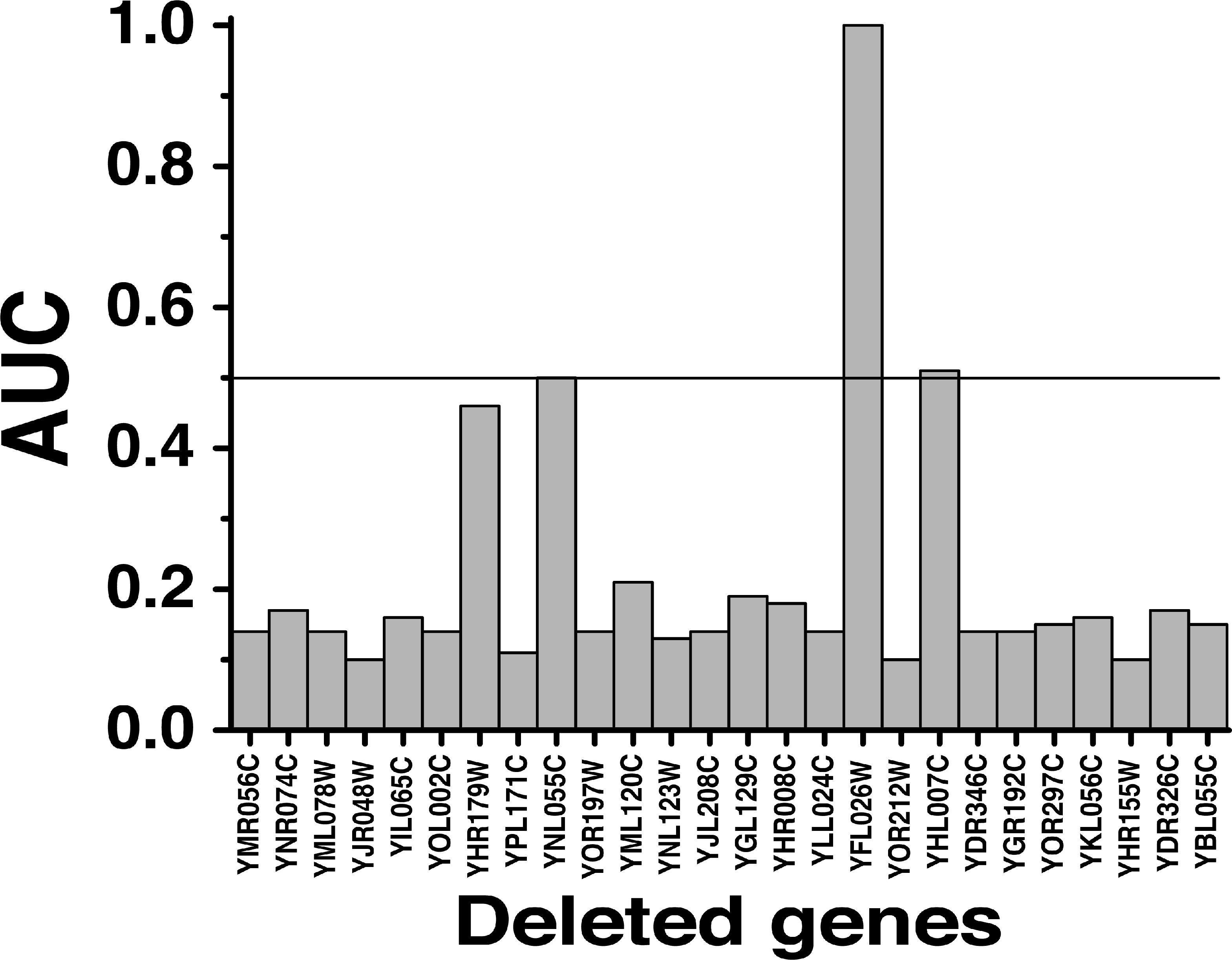

Supplement: Figure S1 — Screening for resistant genes to Iztli peptides. The area under the curve (AUC) for each strain of S. cerevisiae (BY4741) harboring a gene deletion (referred by the ORF name) tested against IP1 (10 µM) was calculated and charted. The AUC control (0.09) is the value of the IP1 (10 µM) against S. cerevisiae WT (BY4741). (TIF) [file pone.0040125.s001.tif]

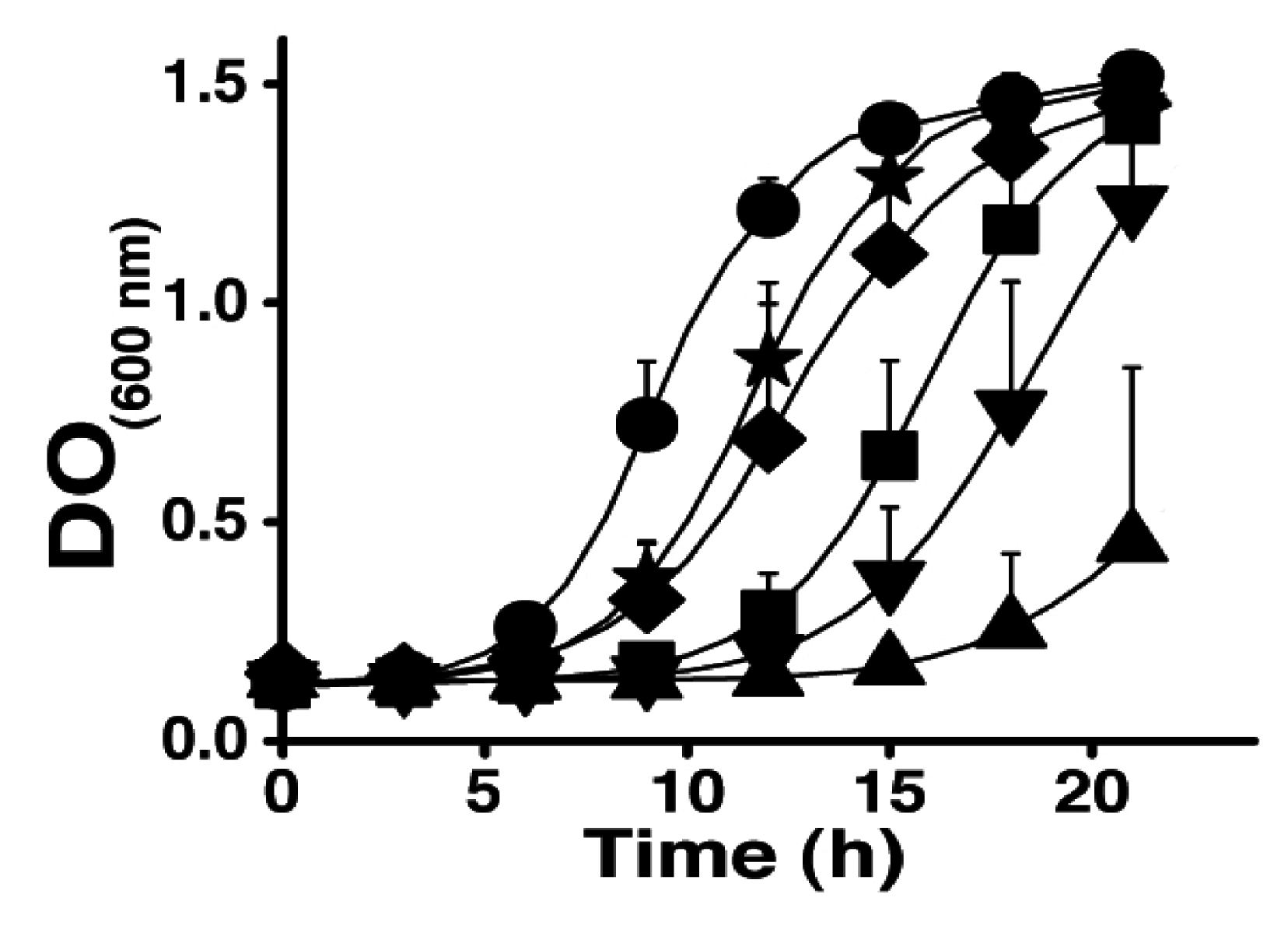

Supplement: Figure S2 — Antifungal activity of Iztli peptides mutated in the recognition sequence. The activity of Iztli peptide 1 mutated in: His8Ala ▪ (2 µM) Gly11Ala ▪ (2 µM), Pro14Ala ▾ (2 µM), Gly15Ala⧫(2 µM) and Tyr19Ala ▪(2 µM) were tested against S. cerevisiae (BY4741), as a control the original IP1 ▪ (2 µM) was used. (TIF) [file pone.0040125.s002.tif]

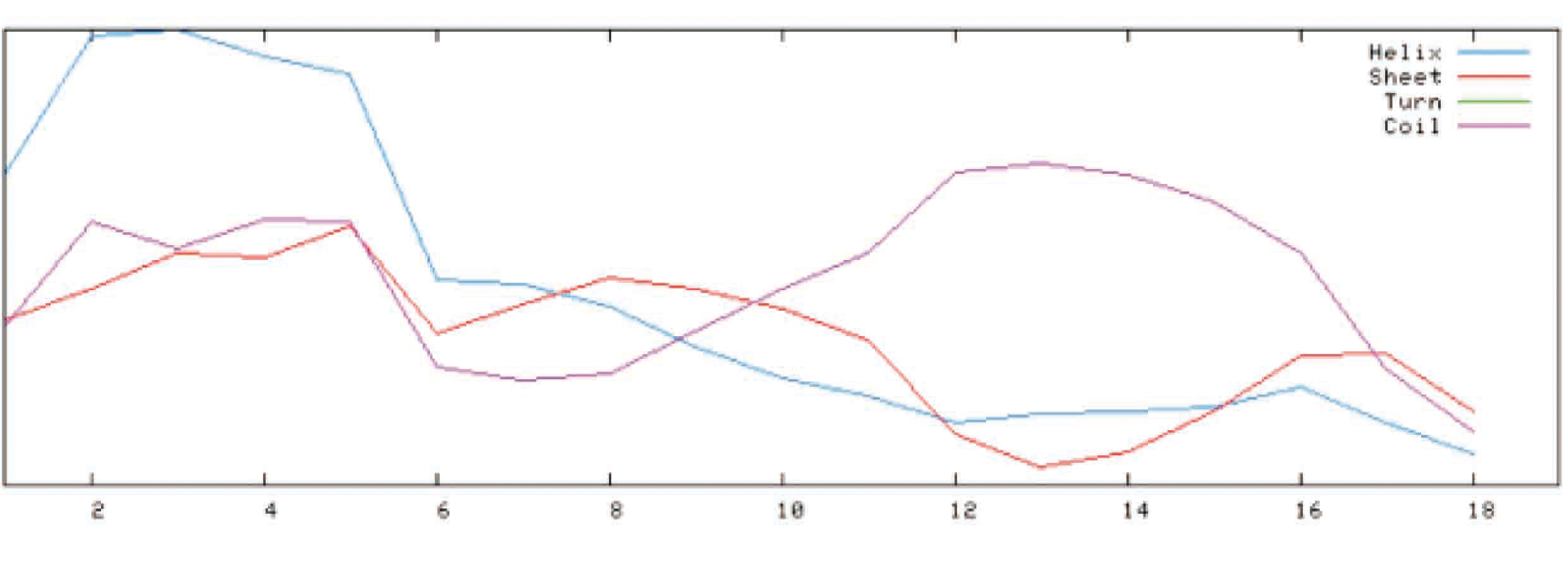

Supplement: Figure S3 — Secondary structure prediction of Iztli Peptides. The SOPMA method was used to generate the image presented [35]. Predictions were generated using the default parameters for SOPMA. Iztli peptides have a larger propensity to form an alpha-helix at the N-terminus and random coil at the C-terminus. (TIF) [file pone.0040125.s003.tif]

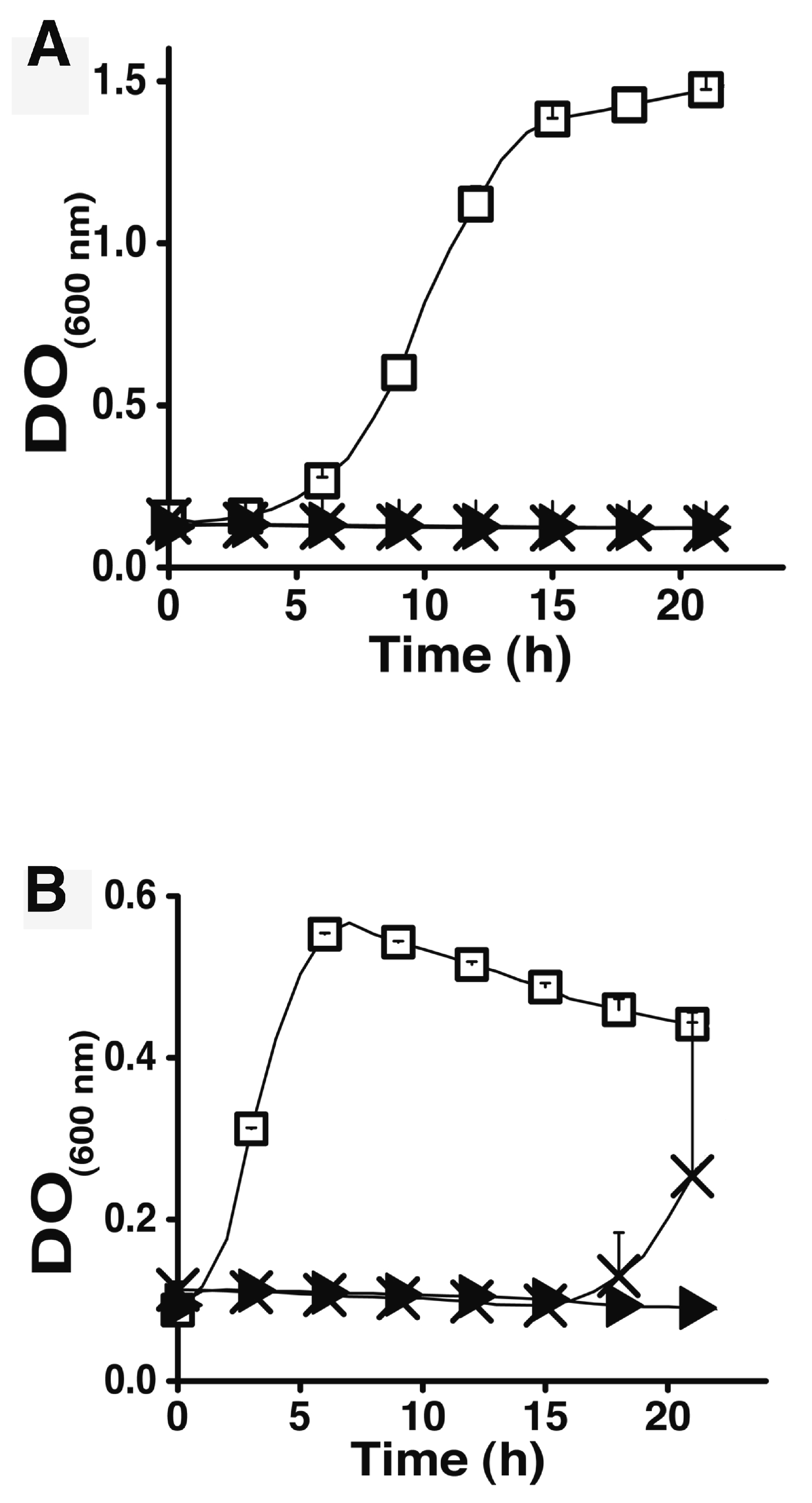

Supplement: Figure S5 — Antifungal and antibacterial activity of IP2SEM. The activity of the seleno methionine derivate of IP2 was tested against A) S. cerevisiae (BY4741), IP2SEM ▪ (10 µM), using like controls YPD without peptide ▪ and IP2 ▪(10 µM) and B) E.coli (DH10B), IP2SEM ▪ (17 µM), like controls were used LB without peptide ▪ and IP2 ▪(17 µM). (TIF) [file pone.0040125.s005.tif]

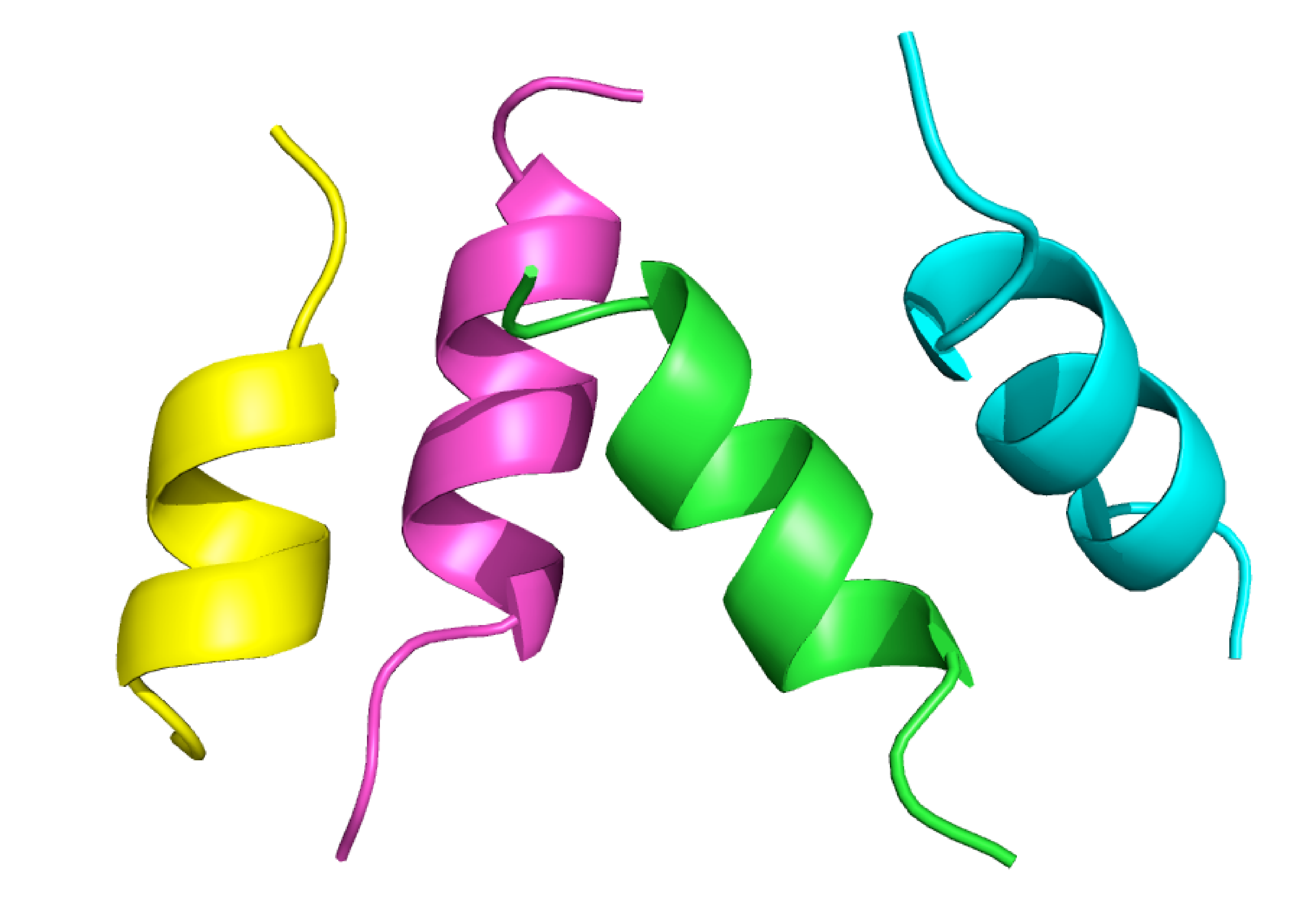

Supplement: Figure S6 — Asymmetric unit of the crystal structure of Iztli peptide 2. The crystal structure of Iztli Peptide 2 was solved at 3.4 Å resolution. Only the alpha-Carbon atoms are presented. The asymmetric unit contains 4 monomers between 11 and 15 residues, each presented as a ribbon of different color. (TIF) [file pone.0040125.s006.tif]

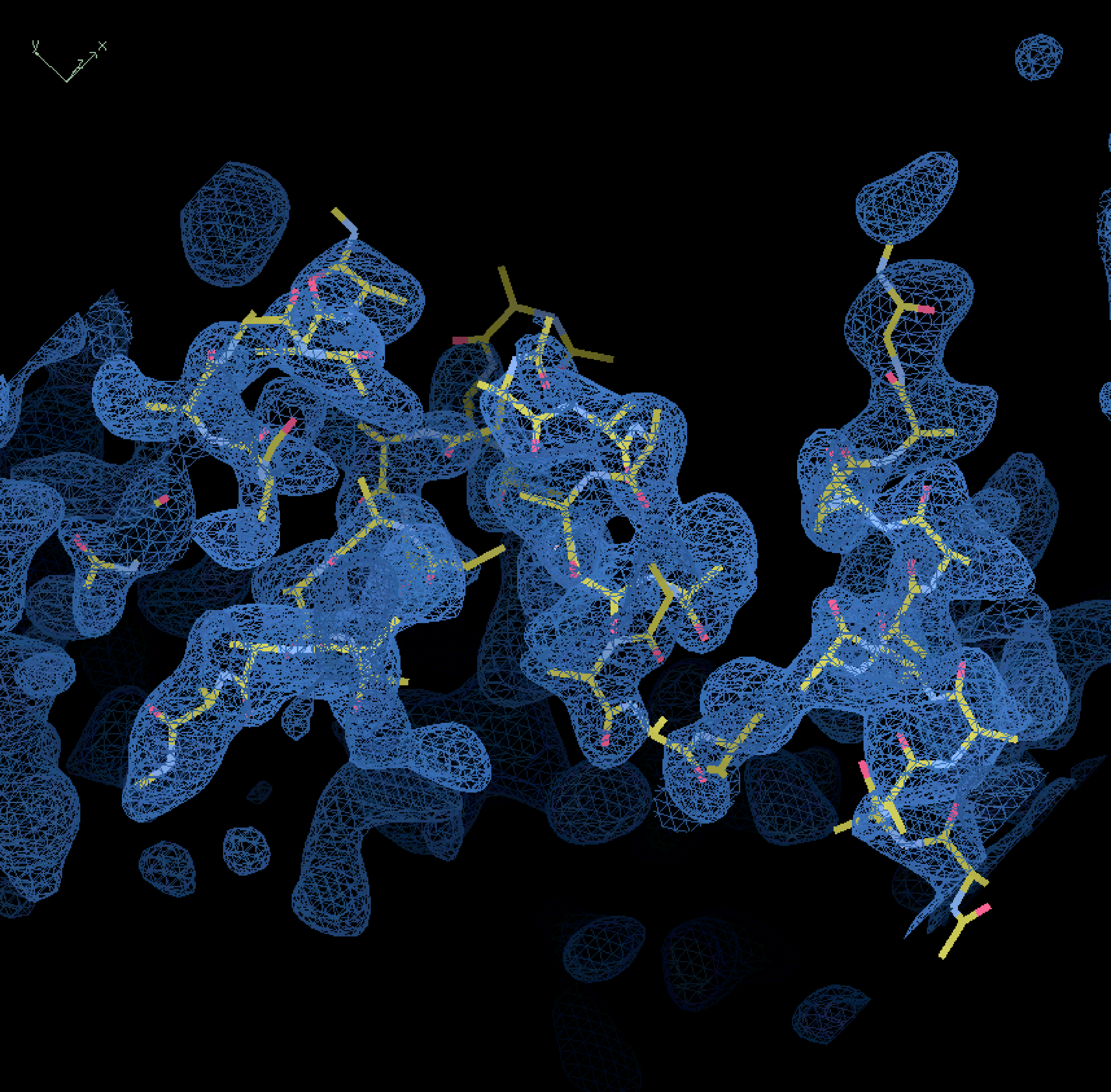

Supplement: Figure S7 — View of the experimental electron density map. Crystal structure of the SeMet derivative Iztli peptide 2. The map is represented as a blue mesh, contoured at 1.4 r.m.s.d. (TIF) [file pone.0040125.s007.tif]

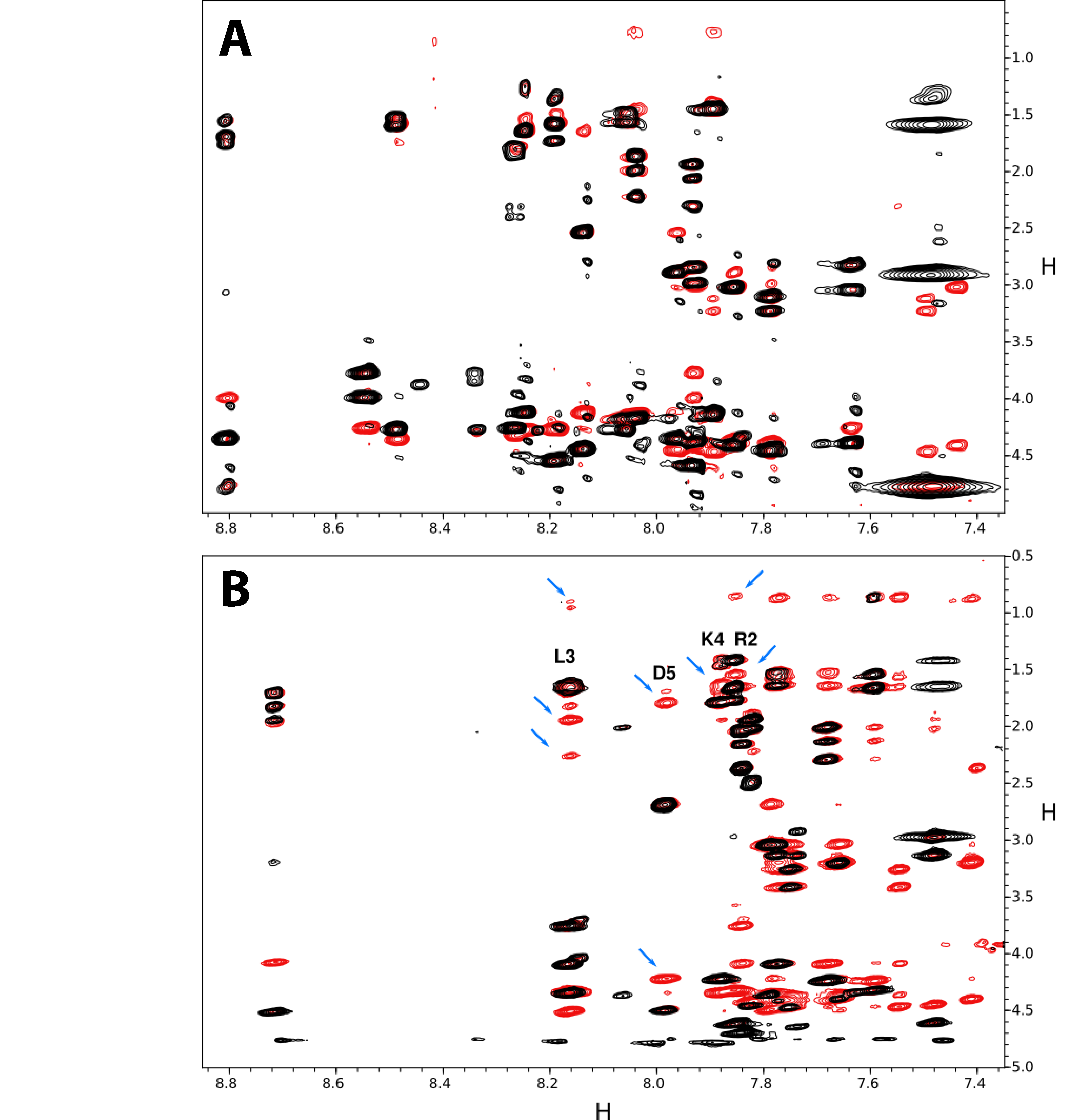

Supplement: Figure S8 — NMR data of IP2. Expanded region of an overlay of 2D-TOCSY (black) and 2D-NOESY (red) spectra of IP2 in H2O a) and 80% TFE b), illustrating the effect of TFE in the inter-residues NOEs cross-peaks. An increase in the number of inter-residues NOEs cross-peaks in 80% TFE is observed and indicated by arrows and one-letter code for the corresponding amino acid residues. The spectra were recorded on a 700 MHz Varian at 298 K. (TIF) [file pone.0040125.s008.tif]

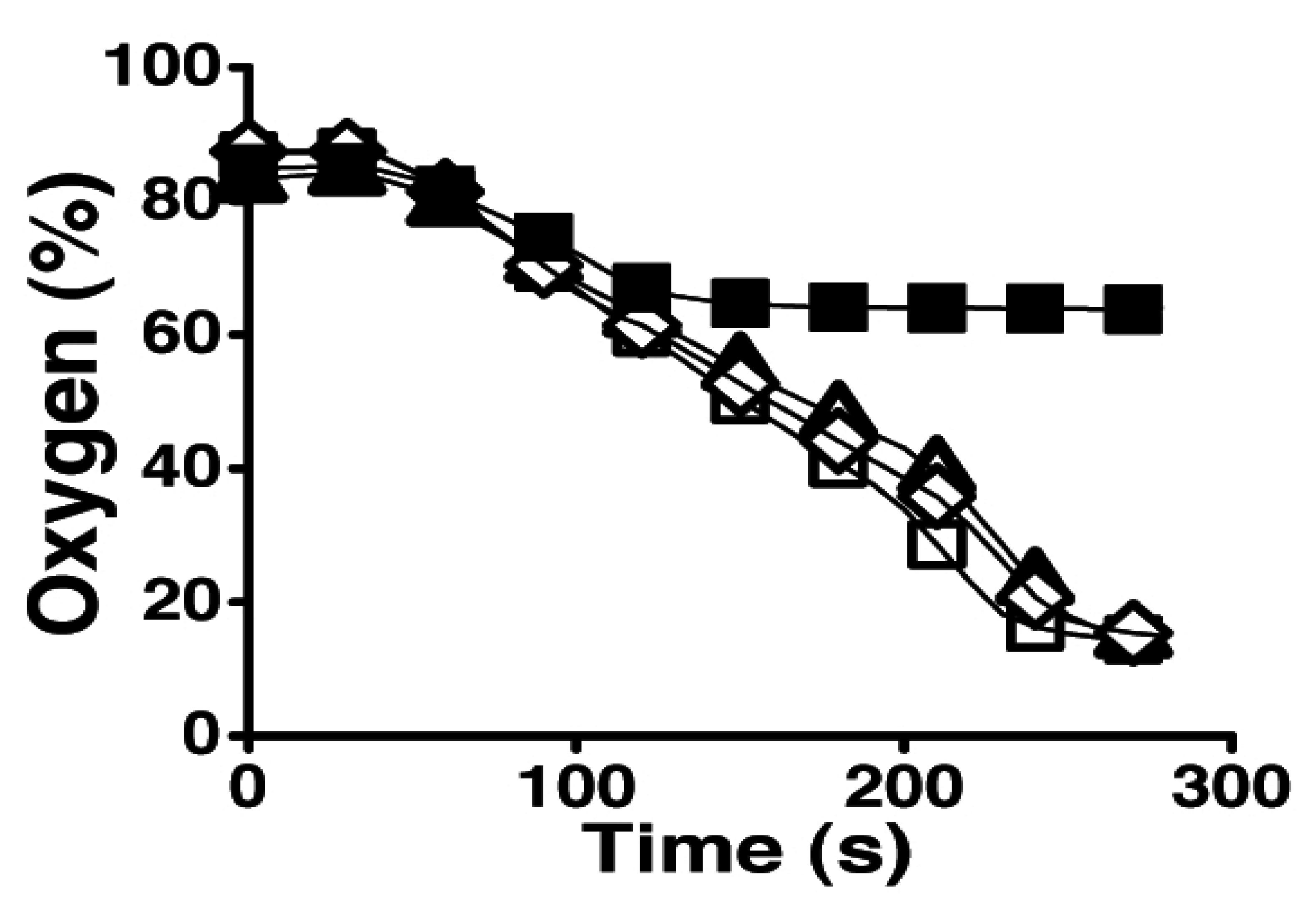

Supplement: Figure S9 — In vivo mitochondrial effect by Iztli peptides. Oxygen consumption of isolated mitochondria of S. cerevisiae (BY4741) was measured in presence of Iztli peptide IP1 ▪ (58 µM) added 80 second after the start of the tracing; as an indicator of respiratory control, CCCP (1 mM final concentration) was added after 180 seconds. For these experiments we used YPD medium without peptide ▪, α-pheromone ▵(10 µM) and fIP1 ⋄ (100 µM) as controls. Iztli peptides IP2, IP3 and IP4 showed a similar effect on mitochondria than IP1. The experiments were repeated three times and the image shows one representative result. (TIF) [file pone.0040125.s009.tif]

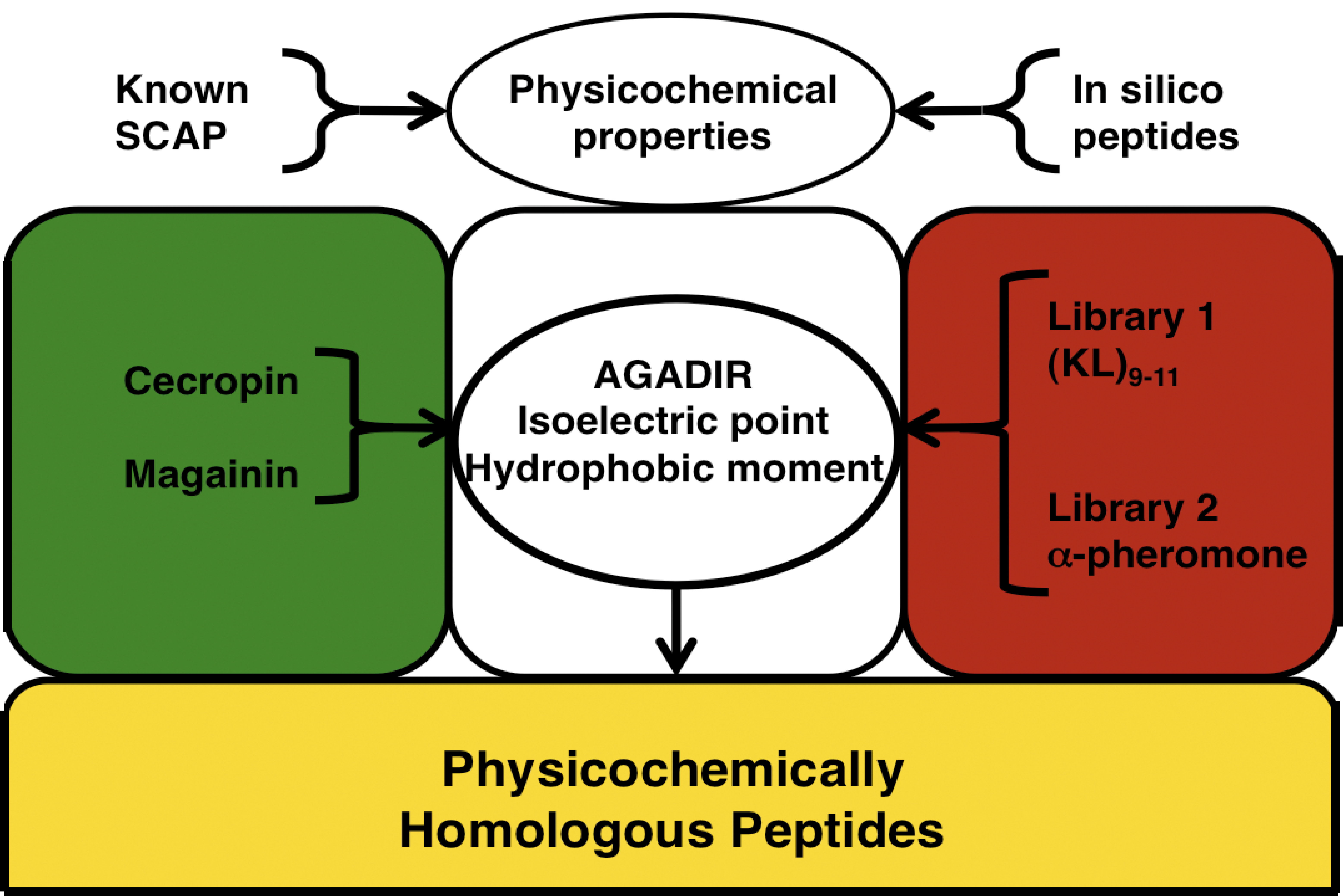

Supplement: Figure S10 — De novo design strategy for selective antibacterial peptides. The strategy described in this work for designing new selective antibacterial peptides is summarized in the figure as follows: a) The data needed for designing SCAP is indicated on top of the scheme. The arrows indicate the relationships between these data: the calculated physicochemical properties of known SCAP are compared with those of peptide sequences generated in silico. b) The specific data used in this study are in the middle of the scheme, that is, the calculated physicochemical properties (AGADIR score, isoelectric point and hydrophobic moment) of Cecropin and Magainin were compared with those obtained from libraries derived from KL and the α-pheromone. c) The new SCAP obtained are indicated at the bottom of the figure (Physicochemically homologous peptides). (TIF) [file pone.0040125.s010.tif]

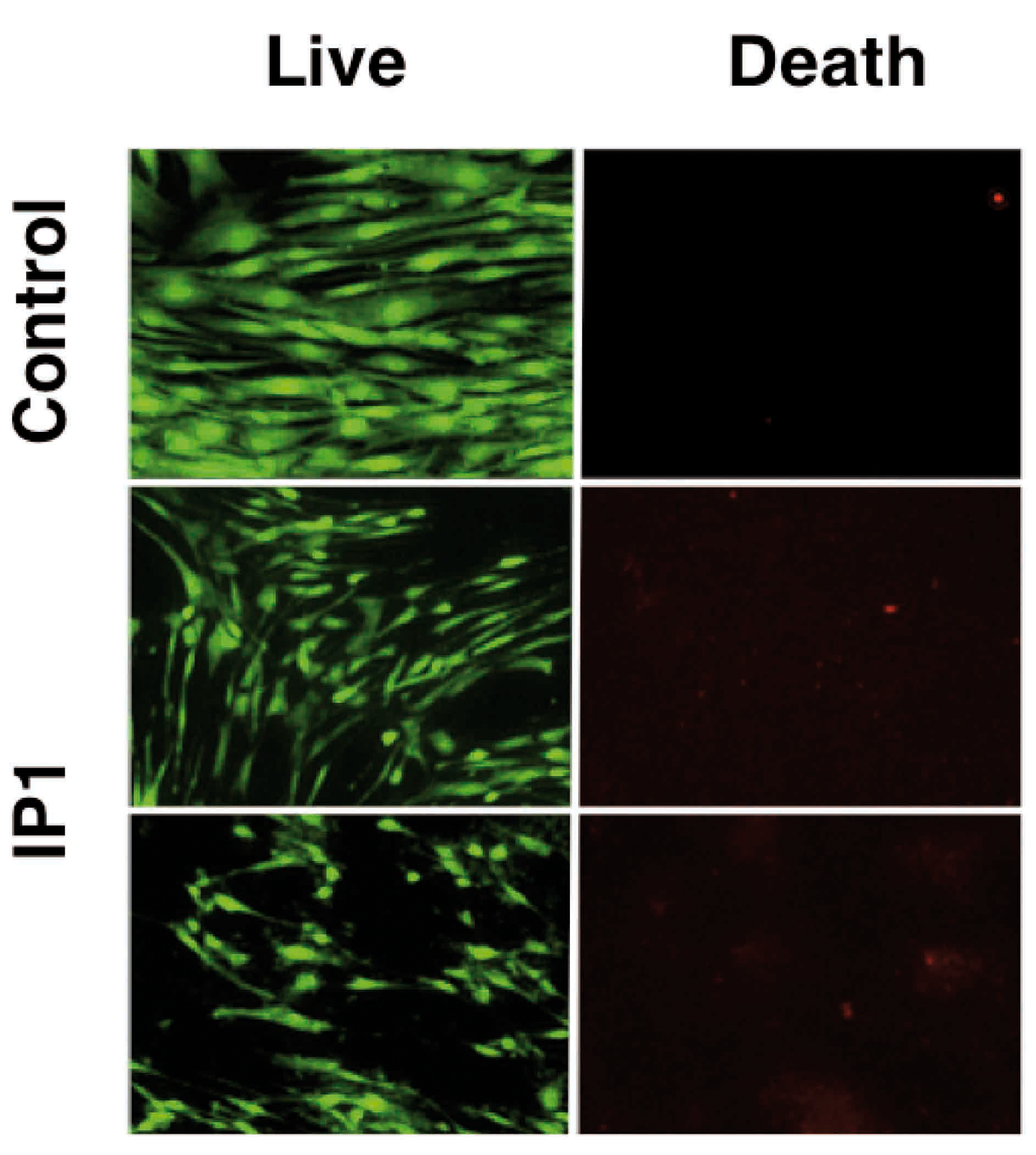

Supplement: Figure S11 — Selected designed SCAP from library 1 are not toxic on HFF cells. The viability of human foreskin fibroblasts (HFF) cells in the presence of water (control), the IP1 and IP2 peptides for 72 hrs are shown. The image shows the living cells stained in green and dead cells stained in red. The images were generated using a magnification of 20×. (TIF) [file pone.0040125.s011.tif]

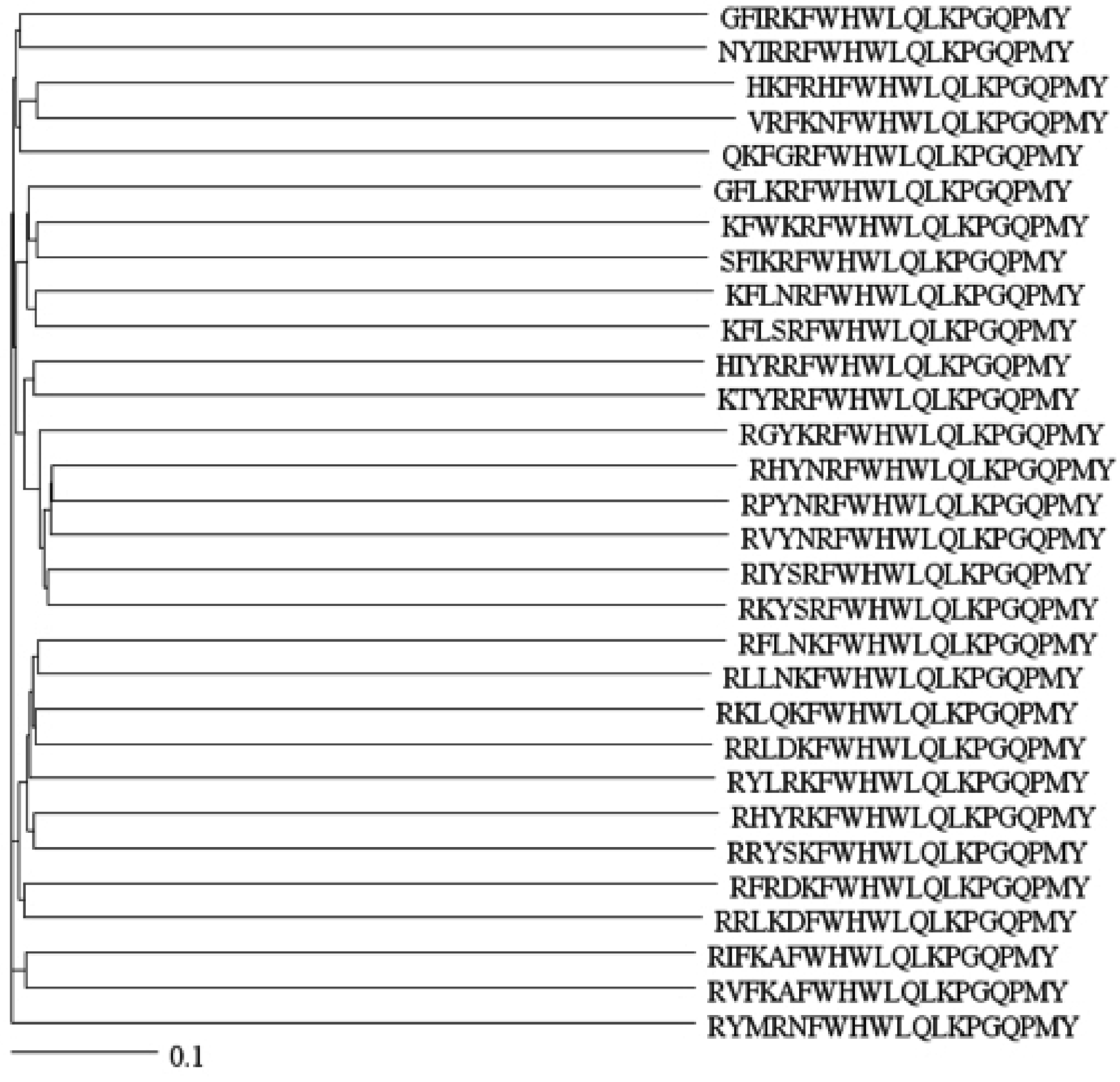

Supplement: Figure S12 — Dendogram of designed SCAP obtained from library 2. The 30 peptide sequences derived from the α-pheromone from S. cerevisiae that were identified as potential SCAP are shown in a dendogram. Each peptide sequence is presented as a node leaf in this representation. (TIF) [file pone.0040125.s012.tif]

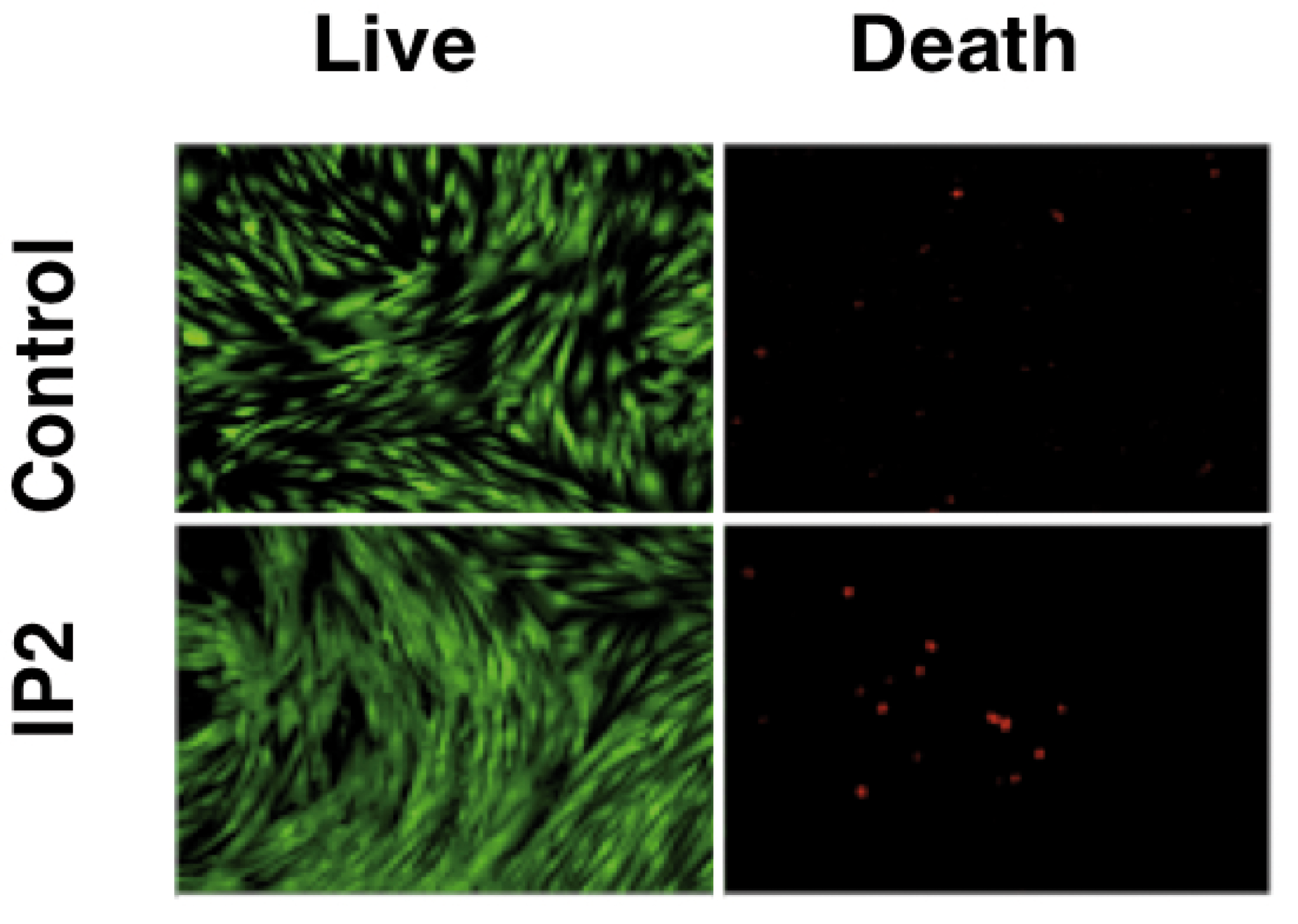

Supplement: Figure S13 — Designed SCAP from library 2 are not toxic on HFF cells. The viability of human foreskin fibroblasts (HFF) cells in the presence of the IP1 or IP2 peptides is shown. The image shows the living cells stained in green and dead cells stained in red. The images were generated using a magnification of 10×. (TIF) [file pone.0040125.s013.tif]

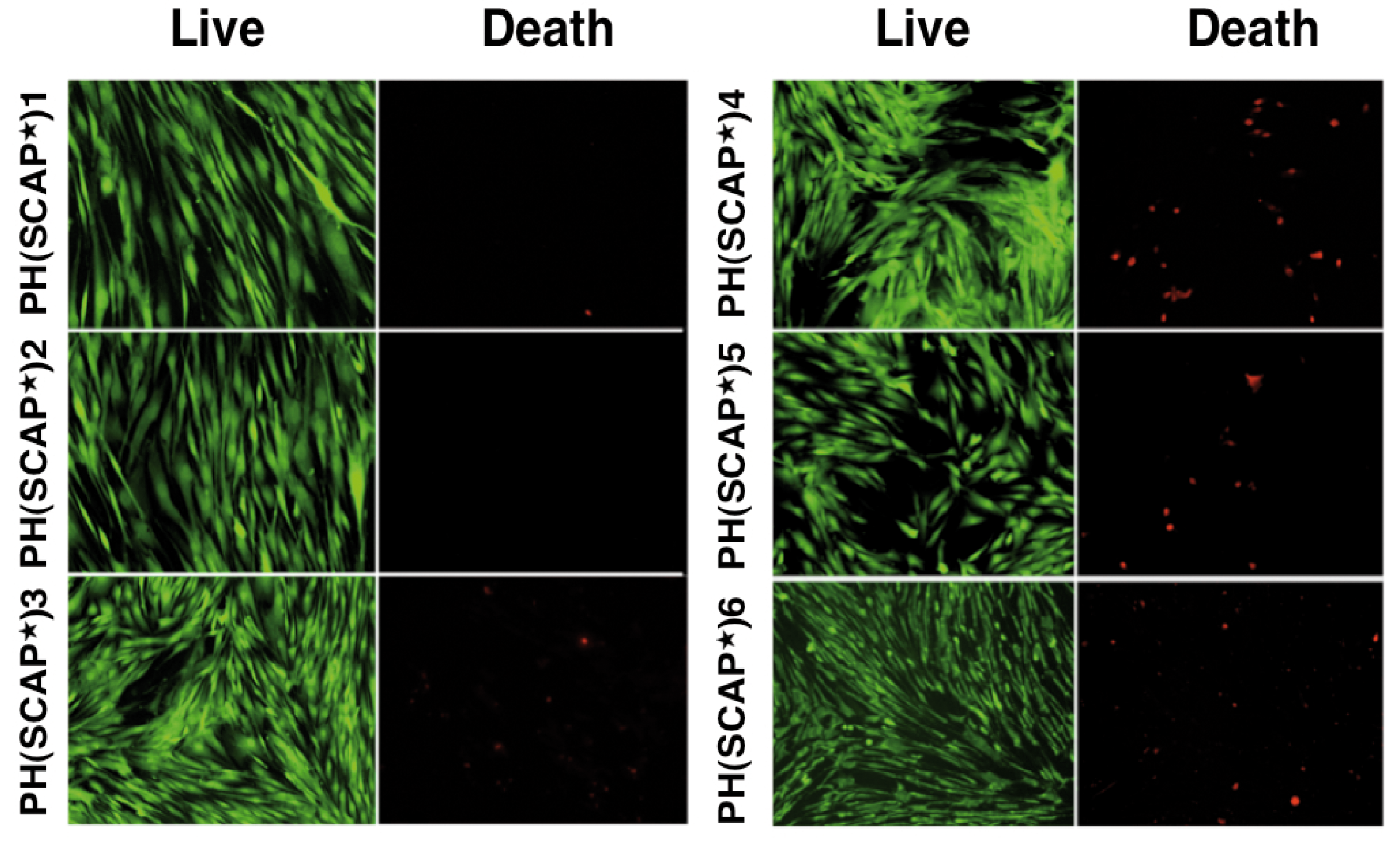

Supplement: Figure S14 — Amphipathic and cationic peptides (control peptides) are not toxic on HFF cells. The viability of human foreskin fibroblasts (HFF) cells in the presence of water and the PH(SCAP*)1, PH(SCAP*)2, PH(SCAP*)3, PH(SCAP*)4, PH(SCAP*)5 and PH(SCAP*)6 peptides are shown. The images were taken 72 hrs after peptide addition (see Methods). The image shows the living cells stained in green and dead cells stained in red. The images were generated with a magnification of 10× for PH(SCAP*)1 and PH(SCAP*)2; 20× for the rest of the peptides. (TIF) [file pone.0040125.s014.tif]

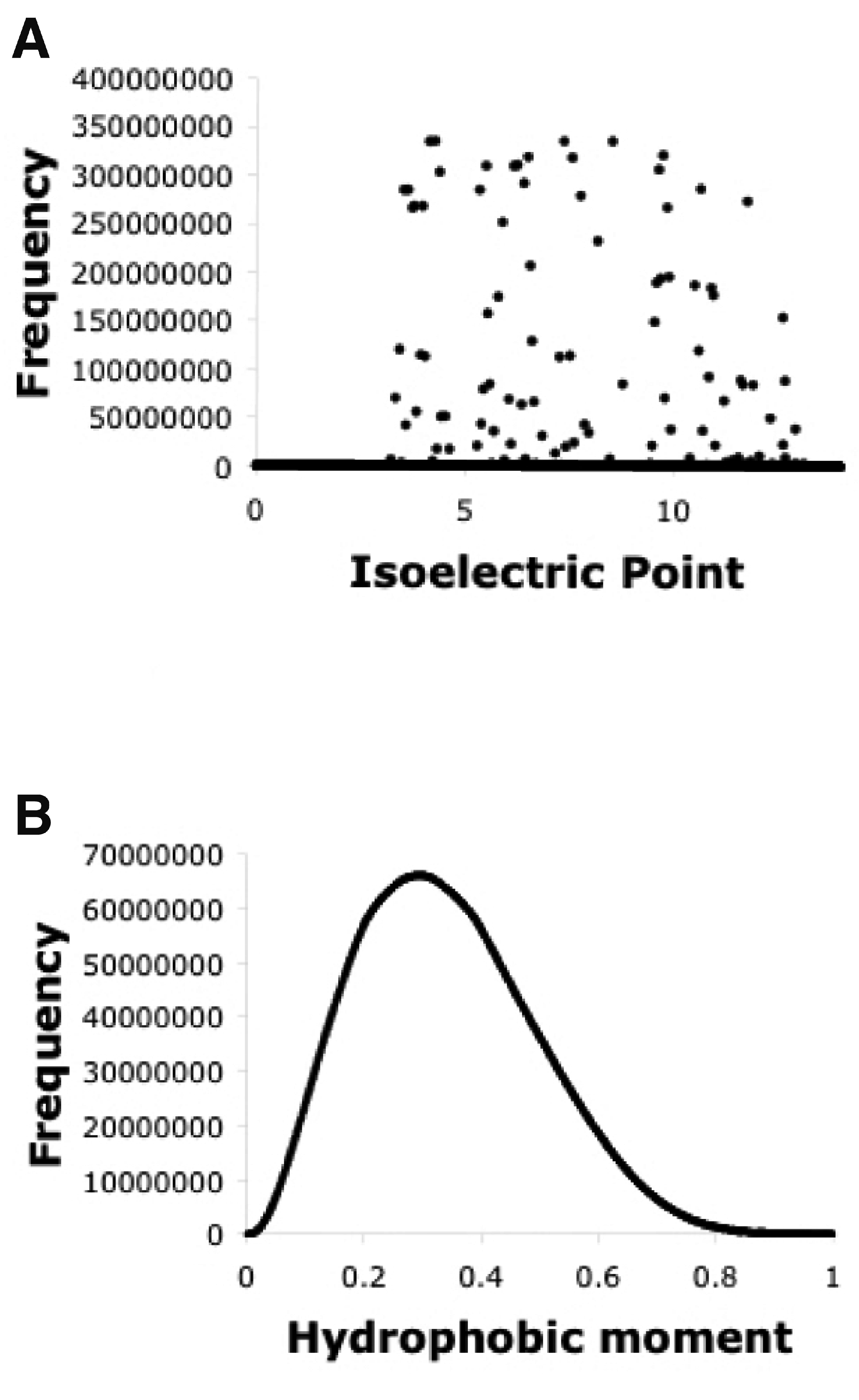

Supplement: Figure S15 — The physicochemical space of peptides. The frequency of predicted values observed in every peptide of length 8 (208 peptides) for A) Isoelectric point and B) hydrophobic moment are displayed. Note that in A) several pH values are not populated (i.e., these have a discrete distribution), while in B) every possible value of hydrophobic moment is found (i.e. these exhibit a continuous distribution). (TIF) [file pone.0040125.s015.tif]

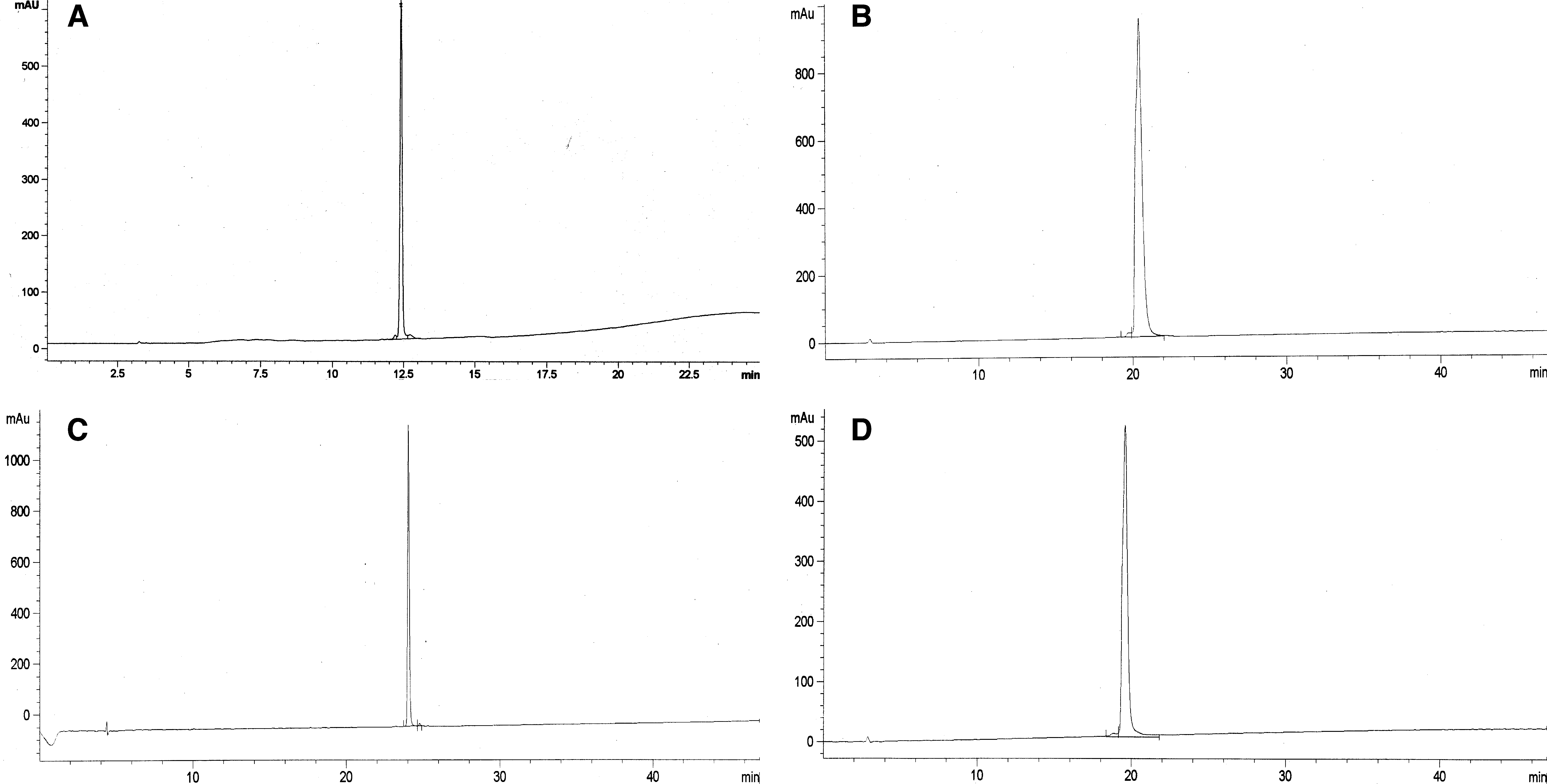

Supplement: Figure S16 — Quality control of Iztli peptides by HPLC. ANASPEC Inc tested each one of the four Iztli peptides reported in this study: A) IP1≥95% purity. B) IP2>95% purity. C) IP3>95% purity. D) IP4>95% purity. (TIF) [file pone.0040125.s016.tif]

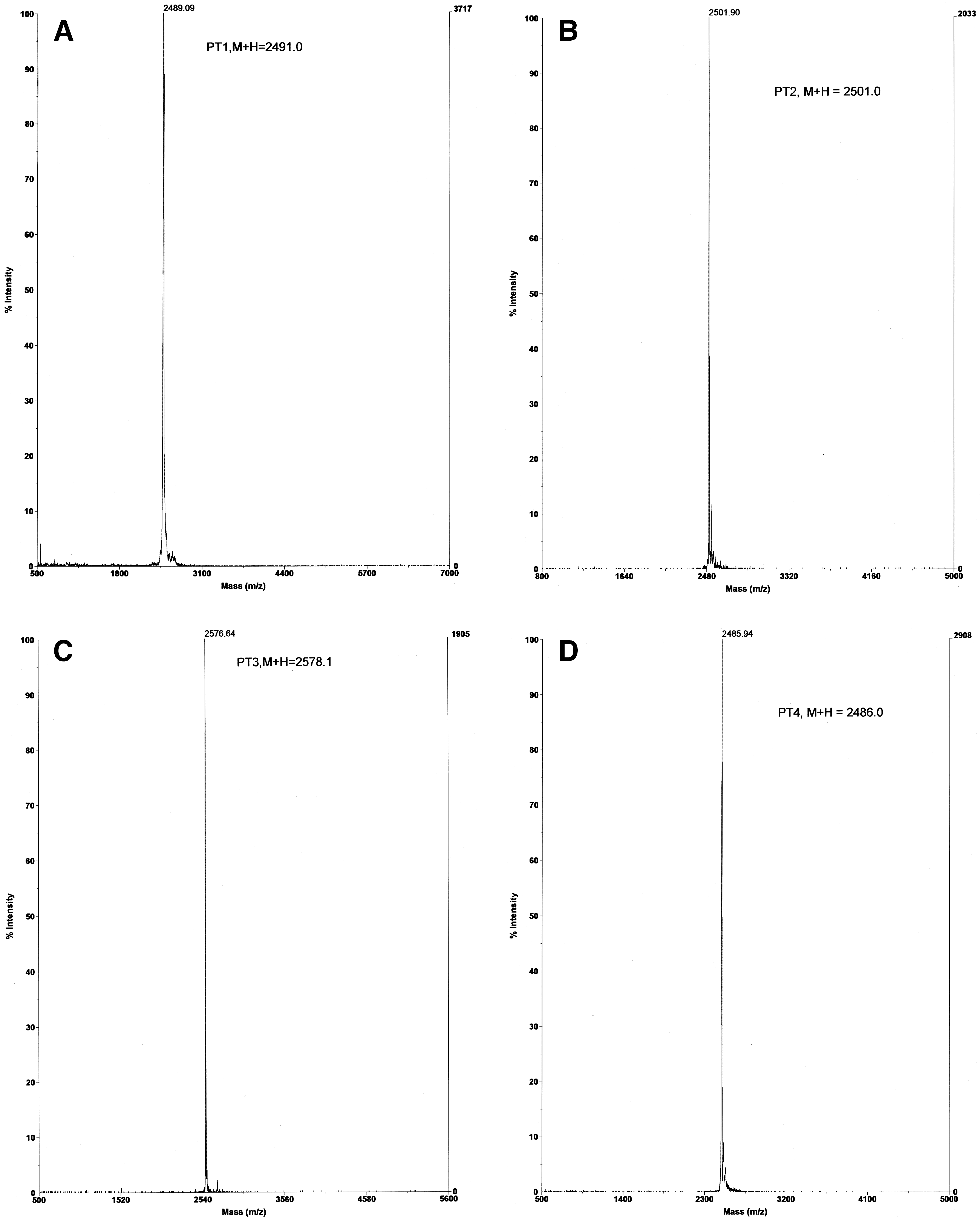

Supplement: Figure S17 — Quality control of Iztli peptides by mass spectrometry. The molecular weight of the four Iztli peptides was determined by ANASPEC Inc. using mass spectrometry: A) IP1∶2490.6 g/mol. B) IP2∶2501.9 g/mol. C) IP3∶2576.6 g/mol. D) IP4∶2485.9 g/mol. (TIF) [file pone.0040125.s017.tif]
