# Supplementary material for: Moonlighting Peptides with Emerging Function
Source: PLoS One. 2012 Jul 13;7(7):e40125. doi: 10.1371/journal.pone.0040125 (PMC3396687; doi:10.1371/journal.pone.0040125)
Supplement: Table S1 — Saccharomyces cerevisiae strains. (DOC) [file pone.0040125.s018.doc]

**Supplementary Table S1. *Saccharomyces cerevisiae* strains**

| Strain | Genotype | Strain | Genotype |
| --- | --- | --- | --- |
| BY4741 | *MATa his3∆1 leu2∆0 met15∆0 ura3∆0* | BY4742 | *MATa his3∆1 leu2∆0*  *lys2∆0 ura3∆0* |
| BY4741 | *MATa ∆atg11* | BY4741 | *MATa ∆pep4* |
| BY4741 | *MATa ∆ayt1* | BY4741 | *MATa ∆por1* |
| BY4741 | *MATa ∆bre2* | BY4741 | *MATa ∆psr2* |
| BY4741 | *MATa ∆cms1* | BY4741 | *MATa ∆saw1* |
| BY4741 | *MATa ∆cox17* | BY4741 | *MATa ∆seo1* |
| BY4741 | *MATa ∆cyc3* | BY4741 | *MATa ∆sir4* |
| BY4741 | *MATa ∆dep1* | BY4741 | *MATa ∆snc1* |
| BY4741 | *MATa ∆dnm1* | BY4741 | *MATa ∆snf7* |
| BY4741 | *MATa ∆drs2* | BY4741 | *MATa ∆spo7* |
| BY4741 | *MATa ∆elm1* | BY4741 | *MATa ∆spo75* |
| BY4741 | *MATa ∆erv46* | BY4741 | *MATa ∆ste20* |
| BY4741 | *MATa ∆frt2* | BY4741 | *MATa ∆ste4* |
| BY4741 | *MATa ∆fun14* | BY4741 | *MATa ∆swc3* |
| BY4741 | *MATa ∆gat3* | BY4741 | *MATa ∆swd1* |
| BY4741 | *MATa ∆isa2* | BY4741 | *MATa ∆syn8* |
| BY4741 | *MATa ∆mht1* | BY4741 | *MATa ∆ubr2* |
| BY4741 | *MATa ∆ntg1* | BY4741 | *MATa ∆uip3* |
| BY4741 | *MATa ∆nup60* | BY4741 | *MATa ∆uth1* |
| BY4741 | *MATa ∆oye2* |  |  |
