# Supplementary material for: Moonlighting Peptides with Emerging Function
Source: PLoS One. 2012 Jul 13;7(7):e40125. doi: 10.1371/journal.pone.0040125 (PMC3396687; doi:10.1371/journal.pone.0040125)
Supplement: Table S2 — Designed selective antibacterial peptides. SCAPs designed in this study are presented indicating their names, sequence, predicted isoelectric point (IP) and helical hydrophobic moment (HM). The observed minimum inhibitory concentration for bacteria (MIC) and human cells (Toxicity) are indicated. For comparison, the SCAP used to match their physicochemical properties (Cecropin, Magainin, CeMa) and the α-pheromone are shown at the bottom of the table. Note that several of these peptides did not show any toxicity against bacteria or mammalian cells (i.e., PH(SCAP*)1.6 and the α-pheromone), therefore the corresponding concentration for the MIC or toxicity are preceded with “bigger than” symbol (>), indicating that possible toxic concentrations should be larger than those tested in this study. ND: not determined. (*) Data obtained from [36]. See section II.1 in this Supporting Information S1 for the rationale on the nomenclature of these peptides. (DOC) [file pone.0040125.s019.doc]

**Supplementary Table S2.  Designed selective antibacterial peptides**

| Name | Sequence | IP | HM | MIC  (μM) | Toxicity  (μM) |
| --- | --- | --- | --- | --- | --- |
| PH(Cecropin)1 | KKLKLLLKLL | 11.3 | 0.44 | 10 | ND |
| PH(Magainin)1 | LKKLLKLLL | 11.1 | 0.56 | 40 | >120 |
| PH(CeMa)1 | LKKLKLLLKLL | 11.3 | 0.43 | 30 | >90 |
| IP1 | KFLNRFWHWLQLKPGQPMY | 11.1 | 0.44 | 20 | >36 |
| IP2 | RRLKDFWHWLQLKPGQPMY | 11.1 | 0.44 | 20 | >36 |
| PH(SCAP*)1 | KWKLFKKI | 11.3 | 0.50 | 153 | >460 |
| PH(SCAP*)2 | GKFLHSAK | 10.8 | 0.42 | >300 | >500 |
| PH(SCAP*)3 | KFLHSAKK | 11.1 | 0.45 | >300 | >500 |
| PH(SCAP*)4 | FLHSAKKF | 10.8 | 0.57 | >300 | >500 |
| PH(SCAP*)5 | HSAKKFGK | 11.1 | 0.40 | >300 | >500 |
| PH(SCAP*)6 | GKFLHSAKKFGK | 11.3 | 0.45 | >300 | >500 |
|  |  |  |  |  |  |
| Cecropin A | KWKLFKKIEKVGQNIRDGIIKAGPAVAVVGQATQIAK | 11.2 | 0.44 | 0.1 (*) | >50 (*) |
| Magainin 2 | GIGKFLHSAKKFGKAFVGEIMNS | 10.8 | 0.56 | 16 (*) | >170 (*) |
| CeMa | KWKLFKKIGIGAVLKVLTTGLPALIS | 11.4 | 0.43 | 0.1 (*) | > 1000 (*) |
| Alpha-Pheromone | WHWLQLKPGQPMY | 9.55 | 0.27 | >18 | ND |

SCAPs designed in this study are presented indicating their names, sequence, predicted isoelectric point (IP) and helical hydrophobic moment (HM). The observed minimum inhibitory concentration for bacteria (MIC) and human cells (Toxicity) are indicated. For comparison, the SCAP used to match their physicochemical properties (cecropin, magainin, CeMa) and the α-Pheromone are shown at the bottom of the table. Note that several of these peptides did not show any toxicity against bacteria or mammalian cells (*i.e*., PH(SCAP*)1..6 and the α-Pheromone), therefore the corresponding concentration for the MIC or toxicity are preceded with “bigger than” symbol (>), indicating that possible toxic concentrations should be larger than those tested in this study. ND: not determined. (*) Data obtained from [3]. See section **II.1** in this supplementary material for the rationale on the nomenclature of these peptides.
