# Supplementary material for: Moonlighting Peptides with Emerging Function
Source: PLoS One. 2012 Jul 13;7(7):e40125. doi: 10.1371/journal.pone.0040125 (PMC3396687; doi:10.1371/journal.pone.0040125)
Supplement: Table S3 — X-ray data collection and refinement statistics. Values in parentheses are for the last resolution shell. (DOC) [file pone.0040125.s020.doc]

**Supplementary Table S3. X-ray data collection and refinement statistics**

**Values in parentheses are for the last resolution shell**

| **Parameters** | **Values** | |
| --- | --- | --- |
| **Data collection statistics** | Native | SeMet |
| Space group | P3_2_12 | P3_2_12 |
| Unit cell dimensions |  |  |
| *a, b, c* (Å) | 37.1, 37.1, 96.6 | 37.5 37.5, 96.3 |
| α, β, γ angles (degrees) | 90.0, 90.0, 120.0 | 90.0, 90.0, 120.0 |
| Resolution range (Å) | 48.3-2.2 (2.32-2.20) | 48.1-3.0 (3.17-3.00) |
| No. of reflections | 14,444 (1,570) | 27,068 (3,681) |
| No. of unique reflections | 3,635 (524) | 1,658 (229) |
| Data completeness (%) | 90.9 (90.7) | 99.2 (98.5) |
| Rsym (%) | 2.3 (22.2) | 3.8 (9.5) |
| I/σ | 11.7 (3.4) | 12.6 (7.9) |
| Mn(I)/sd | 22.3 (3.8) | 53.2 (23.3) |
| No. of peptides in asymmetric unit | 4 | 4 |
| Wilson B-factor (A^2^) | 59.8 | 84.2 |
