# Supplementary material for: Moonlighting Peptides with Emerging Function
Source: PLoS One. 2012 Jul 13;7(7):e40125. doi: 10.1371/journal.pone.0040125 (PMC3396687; doi:10.1371/journal.pone.0040125)
Supplement: Table S4 — Quality control data for Iztli peptide synthesis. Percentage Peak Area reported by ANASPEC Inc. using High-performance liquid chromatography (HPLC) for the four Iztli peptides. (DOC) [file pone.0040125.s021.doc]

**Supplementary Table S4.** Percentage Peak Area reported by ANASPEC Inc. using High-performance liquid chromatography (HPLC) for the four Iztli peptides.

| **Peptide** | **Peak**  **#** | **Ret Time (min)** | **Area [mAU*sec]** | **Height [mAU]** | **Area**  **%** |
| --- | --- | --- | --- | --- | --- |
| IP1 | 1 | 12.207 | 52.042 | 7.500 | 1.4917 |
|  | 2 | 12.418 | 3352.167 | 607.768 | 96.0871 |
|  | 3 | 12.732 | 84.468 | 7.431 | 2.4212 |
| IP2 | 1 | 19.78 | 266.1 | 13.1 | 0.9 |
|  | 2 | 20.41 | 2788-> | 946.1 | 99.1 |
| IP3 | 1 | 24.03 | 9142.2 | 1214.7 | 99.3 |
|  | 2 | 24.76 | 61.1 | 9.4 | 0.7 |
| IP4 | 1 | 19.16 | 197.0 | 6.5 | 1.6 |
|  | 2 | 19.55 | 1207-> | 519.3 | 98.4 |
