# Supplementary material for: Moonlighting Peptides with Emerging Function
Source: PLoS One. 2012 Jul 13;7(7):e40125. doi: 10.1371/journal.pone.0040125 (PMC3396687; doi:10.1371/journal.pone.0040125)
Supplement: Supporting Information S1 — Materials and methods of supplementary figures and tables. The methods used to obtain the reported supplementary figures and tables are included in this document. Also, the corresponding references for these methods are included in this document. (DOC) [file pone.0040125.s022.doc]

**Supporting Online Material for**

**Moonlighting peptides with emerging function**

Jonathan G Rodríguez Plaza, Amanda Villalón Rojas, Sur Herrera, Georgina Garza-Ramos, Alfredo Torres Larios, Carlos Amero, Gabriela Zarraga Granados, Manuel Gutiérrez Aguilar, María Teresa Lara Ortiz, Carlos Polanco Gonzalez, Salvador Uribe Carvajal, Roberto Coria, Antonio Peña Díaz, Dale E. Bredesen, Susana Castro-Obregon, Gabriel del Rio^*^

*To whom correspondence should be addressed. E-mail: [gdelrio@ifc.unam.mx](mailto:gdelrio@ifc.unam.mx)

This document includes

Supplementary Materials and Methods

Supplementary References

**I. Materials and Methods**

**I.1 Measuring Bacterial viability**

The samples were prepared using the same procedure to determine antibacterial activity. However, instead of recording the O.D. in the microplate reader, 5 µL samples of the cells incubated in the presence of the peptides were taken at 0, 6, 12, 18 and 24 hours. These samples were diluted 10^3^, 10^5^, 10^5^, 10^6^ and 10^6^ times respectively, and 5 µL were plated in LB agar plates incubated at 37° C. After 24 hrs the number of colonies were counted for each sample.

**I.2 Mitochondria isolation**

Mitochondria were obtained as described before [1]. Briefly, yeast was incubated in YPD preculture medium for 24 h at 30° C, with agitation at 250 rpm, then the cells were cultured in YPlac at 30° C for 24 h at 250 rpm to improve respiration. Cells were collected and washed twice by centrifugation. The cells were suspended in cold isolating medium (5 mM MES, 0.6 M mannitol, 0.1% bovine serum albumin; the pH was adjusted to 6.8 with triethanolamine). The cells were disrupted into a Bead-Beater cell homogenizer (Biospec Products, USA) with 0.5 mm diameter glass beads, using three 20 seconds pulses separated by 40 seconds intervals. After homogenization, mitochondria were isolated by differential centrifugation. The final mitochondrial pellet was resuspended in 500 μL ice-cold isolation medium. Protein concentration was determined by the Biuret method [2].

**I.3 Toxicity against primary cultures of human cells**

Human foreskin fibroblasts (HFF) cells were grown in high glucose DMEM (Invitrogen, Carlsbad, CA) supplemented with 10% fetal bovine serum (Sigma, St. Louis, MO) and penicillin/streptomycin 100 U/ml (Invitrogen, Carlsbad, CA). The cultures were incubated at 37ºC in 95% air and 5% carbon dioxide with 95% humidity. After 24 hr of plating, 15 micro liters of a stock solution of each peptide was added and 72 hr later cell viability was estimated using the LIVE/DEAD cytotoxicity kit (Molecular Probes L-3224, Invitrogen, Carlsbad, CA) according to manufacturer instructions (4 μM Eth D-1 and 2 μM Calcein AM were used). The esterase substrate calcein AM gives a green fluorescent product when hydrolyzed; therefore, cells stained green have esterase activity and consequently an intact membrane capable of retaining the esterase product. Ethidium homodimer-1, an impermeable dye that binds DNA, enters only into compromised cellular membranes commonly observed in death cells.

Stock solutions of the different peptides were prepared with the following concentrations: PH(Cecropin)1=4 mg/mL, PH(Magainin)1=4 mg/mL, PH(CeMa)1=4 mg/mL, IP1=4.8 mg/mL, IP2=4.8 mg/mL, PH(SCAP*)1= 4 mg/mL, PH(SCAP*)2= 4 mg/mL, PH(SCAP*)3= 12 mg/mL, PH(SCAP*)4= 12 mg/mL, PH(SCAP*)5= 20 mg/mL, PH(SCAP*)6= 12 mg/mL. These concentrations were chosen to be at least 3 times higher than the detected MIC for each peptide. All these concentrations are the dry weight of each peptide.

Images were taken on a Nikon Eclipse TE300 fluorescence microscope from representative fields. Each peptide was tested in two independent assays, each performed in triplicates.

**I.4 Predicted physicochemical properties of peptides**

Three properties were predicted for every sequence peptide analyzed in this study: predicted isoelectric point, hydrophobic moment and AGADIR score. We have coded a program in Fortran77 to perform these calculations (but AGADIR) to facilitate the systematic calculation of these properties. The isoelectric point was calculated based on the following formula:

∑i =10..130(

pH = i/10;

Qtotal = Hi/ (1+10(pH-6.5)) + Ki/ (1+10(pH-10.79)) + Ri/(1+10(pH-12.5)) + 1/ (1+10(pH-8.56)) – [Ci/ (1+10(8.3 -pH)) + Di/(1+10(3.91 -pH)) +Ei/ (1+10(4.25 -pH)) + Yi/ (1+10(10.95 -pH)) + 1/(1+10(3.56 -pH))];

IF (-1/10 < Qtotal < 1/10) THEN pl = pl + pH; i = i+1;

)

where Ci, Di, Ei, Hi, Ki, Ri and Yi are the number of time the amino acids Cystein, Aspartic acid, Glutamic acid, Histidine, Lysine, Arginine and Tyrosine are found in each peptide sequence, respectively. pI is the calculated isoelectric point.

The helical hydrophobic moment was calculated as described previously [3] based on formula described by Eisenberg and collaborators [4] for the hydrophobic moment plot.

**I.5 Computer screening of peptides matching physicochemical properties of known SCAPs**

Two strategies were developed for this purpose. In one strategy, in silico libraries of peptides were generated to reproduce the observed physicochemical properties of a known SCAP, namely Cecropin A, Magainin 2 or a hybrid of Cecropin and Magainin here referred to as CeMa (see Table S2). A peptide sequence was considered to reproduce the predicted isoelectric point of a known SCAP if the values differ in no more than 2%. For instance, the predicted isoelectric point values differing in no more than 0.28 units of pH were considered similar. In the case of the helical hydrophobic moment, a peptide sequence was considered to reproduce the observed hydrophobic moment of a known SCAP only if they have the same value with centesimal precision. This decision was based on the observed distribution of these physicochemical properties (see Figure S15). That is, while almost all possible values for the hydrophobic moment are found in peptide sequences, only few predicted isoelectric values are found in peptide sequences. Finally, peptides differing in less than 1 unit of AGADIR score were considered to have the same AGADIR score.

In another strategy, an in silico library of peptides was produced by fragmenting the Cecropin A or Magainin 2 into peptides of length 8 or 12, in order to identify those having the range of physicochemical properties characteristic of known SCAP [3]: 10.8-11.7 for the predicted isoelectric point, 0.4-0.6 for helical hydrophobic moment and an AGADIR score less than 10.0.

**I.6 Measuring fungal viability**

The samples were prepared using the same procedure as for the determination of antifungal activity. However, instead of recording the O.D. in the microplate reader, 5 µL samples of the cells incubated in the presence of the peptides were taken at 0, 6, 12, 18 and 24 hours. These samples were diluted 10^2^, 10^3^, 10^4^, 10^4^ and 10^4^ times, respectively, and 5 µL were plated in YPD agar plates incubated at 30° C. After 36 hrs the number of colonies for each sample were counted.

**I.7 Identification of critical genes involved in the Iztli peptides activity**

The cultures were prepared as in the antifungal activity assay described before (see above). To identify the strains lacking a gene required to inhibit in at least 50% the normal growth of the cells, the area under the growth curve (AUC) was calculated using software designed ad hoc in our laboratory. Thus, the ratio of the AUCs of the cells in the absence and in the presence of the peptide was determined; any strain rendering a ratio value between 1.0 and 2.0 was considered to carry a deletion of a critical gene.

**I.8 Generation of ρ0 strains of *Saccharomyces cerevisiae***

A colony of *S. cerevisiae* was grown in 2 mL of CSM (Complete Supplement Mix) medium with 5µL of ethidium bromide (Stock solution: 10 mg/mL, sterilized by filtration) at 30° C for 2 days. A sample of 10 µL of this culture was added to 2 mL of new CSM medium with 5 µL of ethidium bromide and incubated at 30° C for 2 days. 10 µL of the culture were streaked in a YPD plate and incubated at 30° C by 36 hours. A colony was streaked in a new YPD plate and incubated at 30° C for 36 hour. To verify the phenotype the plate was replicated with velvet in YPD and YPEG (3% glycerol, 3% ethanol) plates.

**I.9 Mitochondrial respiration**

Oxygen consumption of isolated mitochondria exposed to the Iztli peptides was measured using a YSI model 5300 Oxygraph (Yellow Springs Instrument Co. USA) equipped with a Clark electrode in a 1 mL water jacketed chamber (at 30° C). Such experiments were designed to determine the respiration of mitochondria in its coupled state (state IV). The oxygraph was interfaced to a PC with a voltmeter MUL-600 (Steren, México). Mitochondria (0.5 mg protein/mL) were added to a reaction mixture containing 0.6 M mannitol, 5 mM MES, pH 6.8 (adjusted with triethanolamine), 20 mM KCl, 0.5 mM MgCl, 4 mM Phosphate. Substrate was ethanol 5 μL/mL. Stock solutions were 2.0 M KCl, 1.0 and 1 M Pi–Tris, pH 6.8. CCCP (Carbonyl cyanide m-chlorophenylhydrazone, 5mM) was used to probe that the mitochondrial oxidative phosphorylation coupling, that is, to test for complex IV state. Oxygen consumption was recorded in the absence of peptides for 30 seconds to determine the basal respiration rate of intact mitochondria. After that, the peptides were added at the final concentrations indicated in Figure S9. A zero slope indicates inhibition of the respiration.

**I.10 Crystallization and crystal diffraction of Iztli peptides**

To obtain stable crystals of the Iztli peptides multiple crystallization solutions were tested using the General Screen HT kit (Hampton Research, USA). The peptide was dissolved at a concentration of 32 mg/mL. Crystals were obtained using ammonium sulfate 0.5 M, sodium citrate 100 mM pH 5.6 and lithium sulphate 1 M at 30° C. Crystals were frozen at -173º C with a lithium sulfate 2 M solution. To solve the atomic structure of the Iztli peptides, methionine in the 18th position of Iztli peptide 2 was replaced by a selenomethionine. Data collection was performed at the Life Sciences Collaborative Access Team (LS-CAT) 21-ID-F beamline at the Advance Photon Source (APS) in Argonne, IL, USA. Data processing and integration was performed with XDS (SigAno= 2.644 of the SeMet dataset from the useful range of 60 to 3.4 Å resolution) [5] and MOSFLM [6] and reduced with SCALA [7]. Phases were obtained with the SHELXD program (University of Göttingen, Germany) with the support of Prof. George M. Sheldrick from Göttingen University. We were not able to refine the molecular model obtained (R_free_ stuck at 49.49%) due to a crystal-twinning problem (twin fraction of 0.326). The electron density is extremely poor and it does not allow us to assign the peptide sequence unambiguously (see Figure S7 and Table S3). We are however confident in the alanine molecular model that we were able to provide, as we obtain a molecular replacement solution using PHASER [8] with high Z-scores using the native data (Z-scores for the rotation and translation functions, respectively: 5.9 and 12.9, using the solution with 4 helices in the asymmetric unit obtained from the SeMet derivative).

**I.11 NMR spectroscopy**

All NMR spectra were recorded on a 700 MHz Varian VNMR-S spectrometer equipped with a cryogenically-cooled triple resonance pulsed field gradient probe at the LANEM, in Cuernavaca Mexico. Two-dimensional NMR spectra, NOESY (mixing time 200 ns) and TOCSY (mixing time 75 ms) of the IP2 were recorded in H_2_O and 80% TFE at 298K. All spectra were composed of 8192 complex points in the direct dimension and 1024 complex points in the indirect dimension. All data were processed and analyzed with NMRPipe [9] and CARA [10].

**II. Design of Izlti Peptides**

Iztli peptides are the result of adding a selective antibacterial activity to the α-pheromone; thus, here we describe how to obtain peptides capable to display selective antibacterial activity. We have previously shown that a group of cationic antibacterial peptides (CAPs) display selective action against bacteria. Selective refers to the ability of these peptides not to be toxic to human cells at least at the minimum inhibitory concentration displayed in bacteria. Thus, selective antibacterial peptides here are referred to Selective Cationic Antibacterial Peptides or SCAPs.

Our approach is based on the notion that physicochemical properties determine the SCAPs activity. Thus, any peptide sequence sharing similar physicochemical properties values to known SCAPs may also display SCAP activity (see Figure S10). We refer to these peptides as physicochemically homologous.

To test this idea, two approaches for *de novo* design of SCAPs pharmacophores mimicking the possible mechanisms for the emergence of new genes in nature were used: from pseudo-genes [11] and from highly repetitive sequences [12]. In the last case, we used repeats of Lysine and Leucine residues (see Figure S10, Library 1); in the first case, we used the α-pheromone from *S. cerevisiae* (see Figure S10, Library 2) because it does not have the characteristic physicochemical pattern of SCAPs (see Table S2) nor has any detectable antibacterial activity (see Table S2).

**II.1 Peptide nomenclature**

We assumed the following nomenclature: any peptide designed by physicochemical homology is named using the prefix PH (Physicochemically Homologous), followed by the name of the parent SCAP in parenthesis (in this case, Cecropin A, Magainin 2, CeMa or SCAP* for peptides designed based on every known SCAP), and an integer number. For instance, PH(Cecropin)1 refers to the peptide numbered 1, physicochemically homologous to Cecropin A. In the case where the SCAP was built from an existing peptide (*i.e.*, α-pheromone), the name starts with the name of the existing peptide followed by the PH nomenclature; *e.g*. alpha-pheromone-PH(Cecropin)1 refers to a peptide labeled 1 designed from the α-pheromone that matches the physicochemical properties of Cecropin. For simplicity, alpha-pheromone-PH(Cecropin)1 and alpha-pheromone-PH(CeMa)1 are referred in the main text of this work as Iztli peptide IP1 and IP2, respectively.

**II.2 SCAP from Library 1**

This library included peptides of 9, 10 and 11 residues, with a total of 2^9^ + 2^10^ + 2^11^ = 512+1,024+2,048=3,584 different peptide sequences. From this library only 1 peptide of length 9 matched the properties of Magainin 2, 1 peptide of length 10 matched the properties of Cecropin A and 1 peptide of length 11 matched the properties of the CeMa peptide (see Table S2). These 3 peptides (PH(Cecropin)1, PH(Maginin)1 and PH(CeMa)1; see section II.1 for the rationale on the nomenclature of these peptides) were chemically synthesized and experimentally tested showing antibacterial activity (see Table S2). The toxicity against human cells of peptides PH(Magainin)1 and PH(CeMa)1 was tested; as shown in Figure S11, peptide PH(CeMa)1 formed some precipitates and consequently the cells did not look healthy, but none of the peptides induced cell death detectable by Ethidium homodimer-I staining, and had intact plasma membrane after 72 hr of exposure.

**II.3 SCAP from Library 2**

An *in silico* library was developed that added up to 6 amino acids on each end of the α-pheromone. This library contained a total of 344,857,923 peptide sequences (peptide library’s size = ∑(19i)*(i+1); where i: number of amino acid residues included to the α-pheromone on either extreme; 1,2,3…6). From these, 30 sequences matched the predicted physicochemical values of Cecropin A or CeMa (no matches were found with Magainin 2), and 2 groups were observed based on their sequence similarity (see Figure S12). One peptide sequence from each group was synthesized and experimentally tested for antibacterial activity and toxicity against human cells. We found that peptides IP1 and IP2 presented anti-bacterial activity (see Table S2) and no toxicity against human cells (see Figure S13).

**II.4 SCAP from Control Library**

A library was generated from Cecropin A and Maginin 2 as a control. In this control library, Cecropin and Magainin 2 were fragmented into peptides of 8 to 12 amino acids and new cationic and amphipathic peptides were identified with values of physicochemical properties that are not present in known SCAP. For instance, a peptide may be found in this control library that has a hydrophobic moment value (*e.g*., 0.49) within the observed range of values found in the known SCAP (0.40-0.60), but that particular hydrophobic moment value is not found in any known SCAP (e.g., 0.43, 0.44, 0.56). Note that the length of these peptides include the lengths of those designed from library 1 with SCAP activity (*i.e*., PH(Cecropin)1, PH(CeMa)1 and PH(Magainin)1); such small peptides are unlikely to form helical structures and thus it is possible to expect that any cationic and amphipatic peptide of these lengths would have SCAP activity. Six peptides were detected using this approach (see Table S2 for peptides named PH(SCAP*)1..6). None of these peptides showed any significant antibacterial activity (see Table S2) and none were toxic against human cells (see Figure S14).

**II.5 Distribution of physicochemical properties**

We systematically explored the values of two physicochemical properties of every peptide with 8 amino acid residues (20^8^ = 2.56x10^11^ peptide sequences). Note that our results so far included the use of three properties. However, here we did not use the AGADIR score because we noticed in a random sampling of small peptides (9 residues long), that AGADIR score is always within the range of known SCAP (data not shown). We observed that not every combination of physicochemical values is possible (Figures S15A and S15B). Thus, we considered that any two peptide sequences share identical physicochemical properties values when a) the predicted isoelectric point differ in no more than 2% and b) the predicted hydrophobic moment have the same value with centesimal precision. Finally, peptides differing in less than 1 unit of AGADIR score were considered to have the same AGADIR score.

**Supplementary references**

1. Pena A, Pina MZ, Escamilla E, Pina E (1977) A novel method for the rapid preparation of coupled yeast mitochondria. FEBS Lett 80: 209-213.

2. Gornall AG, Bardawill CJ, David MM (1949) Determination of serum proteins by means of the biuret reaction. J Biol Chem 177: 751-766.

3. del Rio G, Castro-Obregon S, Rao R, Ellerby HM, Bredesen DE (2001) APAP, a sequence-pattern recognition approach identifies substance P as a potential apoptotic peptide. FEBS Lett 494: 213-219.

4. Eisenberg D, Schwarz E, Komaromy M, Wall R (1984) Analysis of membrane and surface protein sequences with the hydrophobic moment plot. J Mol Biol 179: 125-142.

5. Kabsch, W., Automatic processing of rotation diffraction data from crystals of initially unknown symmetry and cell constants. Journal of Applied Crystallography, 1993. 26(6): p. 795-800.

6. Leslie, A.G.W. (1992) Joint CCP4 + ESF-EAMCB Newsletter on Protein Crystallography 26.

7. Collaborative, The CCP4 suite: programs for protein crystallography. Acta Crystallographica Section D, 1994. 50(5): p. 760-763.

8. McCoy, A.J., et al., Phaser crystallographic software. Journal of Applied Crystallography, 2007. 40(4): p. 658-674.

9. Delaglio F, Grzesiek S, Vuister GW, Zhu G, Pfeifer J, et al. (1995) NMRPipe: a multidimensional spectral processing system based on UNIX pipes. J Biomol NMR 6: 277-293.

10. Keller R (2004) The computer Aided Resonance Assignment; Verlag C, editor.

11. Begun DJ (1997) Origin and evolution of a new gene descended from alcohol dehydrogenase in Drosophila. Genetics 145: 375-382.

12. Li YC, Korol AB, Fahima T, Nevo E (2004) Microsatellites within genes: structure, function, and evolution. Mol Biol Evol 21: 991-1007.
